# Neonatal and maternal adverse outcomes and exposure to nonsteroidal anti-inflammatory drugs during early pregnancy in South Korea: A nationwide cohort study

**DOI:** 10.1371/journal.pmed.1004183

**Published:** 2023-02-27

**Authors:** Eun-Young Choi, Han Eol Jeong, Yunha Noh, Ahhyung Choi, Dong Keon Yon, Jung Yeol Han, Ji-Hee Sung, Seung-Ah Choe, Ju-Young Shin

**Affiliations:** 1 School of Pharmacy, Sungkyunkwan University, Suwon, South Korea; 2 Department of Biohealth Regulatory Science, Sungkyunkwan University, Suwon, South Korea; 3 Department of Pediatrics, Kyung Hee University Medical Center, Kyung Hee University College of Medicine, Seoul, South Korea; 4 Center for Digital Health, Medical Science Research Institute, Kyung Hee University College of Medicine, Seoul, South Korea; 5 Korean Mothersafe Counselling Center, Department of Obstetrics and Gynecology, Inje University Ilsan Paik Hospital, Goyang, South Korea; 6 Department of Obstetrics and Gynecology, Samsung Medical Center, Sungkyunkwan University School of Medicine, Seoul, South Korea; 7 Department of Preventive Medicine, Korea University College of Medicine, Seoul, South Korea; 8 Samsung Advanced Institute for Health Sciences & Technology, Sungkyunkwan University, Seoul, South Korea; N/A, UNITED KINGDOM

## Abstract

**Background:**

Existing data on the use of nonsteroidal anti-inflammatory drugs (NSAIDs) during late pregnancy is well established, providing assurance. However, the use of NSAIDs during early pregnancy remains inconclusive owing to conflicting findings on adverse neonatal outcomes as well as the limited data on adverse maternal outcomes. Therefore, we sought to investigate whether early prenatal exposure to NSAIDs was associated with neonatal and maternal adverse outcomes.

**Methods and findings:**

We conducted a nationwide, population-based cohort study using Korea’s National Health Insurance Service (NHIS) database with a mother–offspring cohort constructed and validated by the NHIS to include all live births in women aged 18 to 44 years between 2010 and 2018. We defined exposure to NSAIDs as at least two records of NSAID prescriptions during early pregnancy (first 90 days of pregnancy for congenital malformations and first 19 weeks for nonmalformation outcomes) and compared against three distinct referent groups of (1) unexposed, no NSAID prescription during the 3 months before pregnancy start to end of early pregnancy; (2) acetaminophen-exposed, at least two acetaminophen prescriptions during early pregnancy (i.e., active comparator); and (3) past users, at least two NSAID prescriptions before the start of pregnancy but no relevant prescriptions during pregnancy. Outcomes of interest were adverse birth outcomes of major congenital malformations and low birth weight and adverse maternal outcomes of antepartum hemorrhage and oligohydramnios. We estimated relative risks (RRs) with 95% CIs using generalized linear models within a propensity score (PS) fine stratification weighted cohort that accounted for various potential confounders of maternal sociodemographic characteristics, comorbidities, co-medication use, and general markers of burden of illness. Of 1.8 million pregnancies in the PS weighted analyses, exposure to NSAIDs during early pregnancy was associated with slightly increased risks for neonatal outcomes of major congenital malformations (PS-adjusted RR, 1.14 [CI, 1.10 to 1.18]) and low birth weight (1.29 [1.25 to 1.33]), and for maternal outcome of oligohydramnios (1.09 [1.01 to 1.19]) but not antepartum hemorrhage (1.05 [0.99 to 1.12]). The risks of overall congenital malformations, low birth weight, and oligohydramnios remained significantly elevated despite comparing NSAIDs against acetaminophen or past users. Risks of adverse neonatal and maternal outcomes were higher with cyclooxygenase-2 selective inhibitors or use of NSAIDs for more than 10 days, whereas generally similar effects were observed across the three most frequently used individual NSAIDs. Point estimates were largely consistent across all sensitivity analyses, including the sibling-matched analysis. Main limitations of this study are residual confounding by indication and from unmeasured factors.

**Conclusions:**

This large-scale, nationwide cohort study found that exposure to NSAIDs during early pregnancy was associated with slightly higher risks of neonatal and maternal adverse outcomes. Clinicians should therefore carefully weigh the benefits of prescribing NSAIDs in early pregnancy against its modest, but possible, risk of neonatal and maternal outcomes, where if possible, consider prescribing nonselective NSAIDs for <10 days, along with continued careful monitoring for any safety signals.

## Abstract 2

Eun-Young Choi and colleagues investigate whether early prenatal exposure to non-steroidal anti-inflammatory drugs was associated with adverse neonatal and maternal outcomes in South Korea.

## Author summary

**Why was this study done?:**

- Fever, pain, and inflammation are not uncommon during pregnancy, with nonsteroidal anti-inflammatory drugs (NSAIDs) being a possible treatment option for pregnant women to attenuate these conditions.
- Possible risks of adverse neonatal and maternal outcomes following exposure to NSAIDs during early pregnancy remain inconclusive and uncertain to date.

**What did the researchers do and find?:**

- In this large-scale, nationwide, population-based cohort study of 1.8 million pregnancies, exposure to NSAIDs in early pregnancy was significantly associated with 14%, 29%, and 9% higher risks of major congenital malformations, low birth weight, and oligohydramnios, respectively, when compared with unexposed pregnancies.
- Stronger positive associations were found between neonatal and maternal adverse outcomes and the use of COX-2 inhibitors or use of NSAIDs for more than 10 days during early pregnancy.

**What do these findings mean?:**

- Given the limited treatment option available for pregnant women, clinicians should carefully weigh the benefits of prescribing NSAIDs in early pregnancy against its modest, but possible, risk of neonatal and maternal outcomes.
- Clinicians and pregnant women should consider prescribing or receiving nonselective NSAIDs for <10 days, respectively, with continued careful monitoring for any safety signals.

## Introduction

Fever, pain, and inflammation during pregnancy are not uncommon and complex conditions to manage, where if managed poorly, these may result in maternal complications [1]. Accordingly, nonsteroidal anti-inflammatory drugs (NSAIDs) are a possible treatment option for pregnant women to treat and/or attenuate these conditions, and indications for chronic use during pregnancy are inflammatory bowel or chronic rheumatic diseases [2,3]. However, NSAIDs may increase the risk of embryo-fetal adverse effects, dependent on the type of NSAIDs, period of gestation, and duration of consumption, due to its mechanism of action (e.g., inhibition of prostanoid activity) [3–6]. Despite these concerns, exposure to NSAIDs during early pregnancy is not infrequent by either being unaware of the pregnant state or simply due to the extremely limited treatment option available for pregnant women, albeit the inconclusive data on its safety [7,8].

Unlike the relatively established risks of premature closure of the ductus arteriosus and oligohydramnios with NSAID use during late pregnancy [9–14], existing data on early prenatal NSAID use and the risk of adverse neonatal outcomes were largely based on self-reported use, outdated, had a small sample size, or did not control for confounding by indication (S1 **Table**) [10,15–19]. Thus, evidence on the use of NSAIDs during early pregnancy remains unclear, with particular knowledge gaps present on cyclooxygenase (COX)-2 inhibitors [3,20]. The association between prenatal NSAID use and obstetric complications is even more scarce. Hence, further studies with sufficient sample size and advanced epidemiologic methods are needed to investigate into the currently indeterminate association of adverse birth outcomes, including major malformations, and obstetric complications with early prenatal exposure to NSAIDs.

Being aware of the underlying risk associated with prenatal NSAID use is highly important given the substantial number of women exposed to these medications and the very limited option of analgesics present for use during pregnancy [17,21,22]. There is also a strong need for a well-designed, real-world evidence to assist pregnant women or women of childbearing age and their healthcare providers to weigh the risks of using NSAIDs against its benefits during this physiologically complex period [23]. This study was therefore aimed to examine the association between early prenatal exposure to NSAIDs and the risk of adverse neonatal and maternal outcomes by using a nationwide mother–child linked cohort in South Korea.

## Methods

### Data source

Using South Korea’s National Health Insurance Service-National Health Insurance Database (NHIS-NHID) [24], we identified all pregnancies in women aged 18 to 44 years with live births between April 1, 2010, and December 31, 2018. The mother–offspring cohort was constructed and provided solely for research purposes by the NHIS, which used a self-developed linkage algorithm based on the unique health insurance card number shared within a family and the date of delivery; this algorithm has been internally validated by the NHIS. In brief, the NHIS-NHID includes comprehensive information on sociodemographic variables, in- and outpatient healthcare utilization including diagnoses, procedures, and prescriptions, health examination records (for both mothers and their offspring), and vital statistics linked from Statistics Korea.

The study protocol was approved by the Institutional Review Board of Sungkyunkwan University (2021-04-024), and all analyses were conducted according to this protocol (**S1 Protocol**); informed patient consent was not required as our study used deidentified health insurance claims data.

### Study cohorts

As the etiologically relevant exposure window, the outcome assessment window, and the covariate assessment window differ by study outcome, we created two separate study cohorts. First, cohort 1 was constructed for the analysis of congenital malformations by setting the window of interest between the last menstrual period (LMP) to LMP+90 (hereafter, first trimester), and we excluded the following: (1) pregnancies with exposure to known teratogenic drugs (e.g., antineoplastic agent, warfarin, lithium, systemic retinoids, misoprostol, thalidomide, androgens, antiepileptic medications [valproate, topiramate, carbamazepine, oxcarbazepine, phenobarbital, phenytoin]); (2) infants with chromosomal abnormalities, genetic syndromes, and malformation syndromes with known causes; (3) pregnancies with no NSAID prescription during the first trimester, but with ≥1 NSAID prescription within 3 months before the LMP (LMP-90 to LMP-1); and (4) pregnancies with only 1 NSAID prescription during the first trimester to minimize potential for misclassification of exposure. Second, cohort 2 was constructed for the analysis of nonmalformation outcomes of low birth weight, antepartum hemorrhage, and oligohydramnios, by setting a broader window to evaluate the effects of exposure prior to the outcome assessment period (LMP to 19 week of gestation [hereafter, early pregnancy]), where we excluded (1) pregnancies with no NSAID prescription during early pregnancy, but with ≥1 NSAID prescription within 3 months before the LMP, and (2) pregnancies with only 1 NSAID prescription during early pregnancy.

### Exposure

We considered pregnant women prescribed ≥2 NSAIDs during the etiologically relevant window to be defined as those exposed to NSAIDs; if a woman refilled her prescription for NSAIDs, we assumed that she probably took them. The etiologically relevant window for major congenital malformations (cohort 1) was defined as the first trimester and for low birth weight, antepartum hemorrhage, and oligohydramnios (cohort 2) as early pregnancy. Pregnancies were considered unexposed if not prescribed any NSAIDs from 90 days before the LMP throughout the end of the first trimester or end of early pregnancy for cohorts 1 and 2, respectively. The LMP date was estimated by using a previously validated algorithm to estimate gestational age in administrative healthcare databases [25].

### Outcomes

Major congenital malformations were defined as infants in the first year of life with major congenital malformations according to the classification system of the European Surveillance of Congenital Anomalies (EUROCAT) subgroups of major congenital anomalies [26,27]. The presence of congenital malformations was defined using previously validated algorithms based on inpatient or outpatient diagnostic and procedural codes, which have shown to identify the outcomes with high specificity (i.e., ≥1 date with the respective diagnostic code or 1 diagnostic code and a code for infant death between delivery and 1 year after delivery) [28,29]. We excluded major congenital malformations with known causes and minor defects according to the EUROCAT exclusion list. Major congenital malformations were also categorized into 12 organ-specific malformations: (1) nervous system; (2) eye; (3) ear, face, and neck; (4) heart defects; (5) respiratory system; (6) oral clefts; (7) digestive system; (8) abdominal wall defects; (9) urinary system; (10) genital organs; (11) limb; and (12) other malformations. Nonmalformation outcomes of interest were low birth weight, antepartum hemorrhage, and oligohydramnios. We defined low birth weight using neonatal inpatient or outpatient diagnosis claims recorded within 30 days of delivery (International Classification of Disease, 10th Revision: P07) and used maternal inpatient or outpatient diagnosis claims recorded from week 20 of gestation to delivery to identify antepartum hemorrhage (O44.1, O46) and oligohydramnios (O41.0). Detailed definitions for each outcome available in **S2 Table.**

### Covariates

We assessed a broad range of potential confounders: maternal sociodemographic characteristics (e.g., maternal age, income level, parity, multiple gestations), comorbidities (e.g., gastrointestinal diseases, fever), co-medication use (e.g., antiepileptics, opioid analgesics), and general markers of burden of illness (e.g., obstetric comorbidity index; [30,31]). Maternal characteristics were measured at delivery, and comorbidities and co-medication use were measured from 6 months before pregnancy to end of the first trimester for cohort 1 and to the end of early pregnancy for cohort 2. Measures of healthcare utilization were assessed during the 6 months before but not during pregnancy (LMP-180 to LMP-1), such that these measures were unaffected by the early detection of pregnancy complications.

### Statistical analyses

#### Primary analysis

We described baseline characteristics for each cohort, comparing NSAID-exposed versus unexposed pregnancies, and assessed between-group covariate balance using standardized mean differences (SMDs), considering its absolute value ≥0.1 as important imbalances [32]. For all outcomes, we estimated its absolute risk per 1,000 births or pregnancies. To adjust for underlying differences between groups, we derived propensity scores (PS) from the predicted probability of being exposed to NSAIDs using a multivariable logistic regression model that included all covariates mentioned above as independent variables. We excluded observations in nonoverlapping regions of the PS distributions and identified 50 equally sized PS strata based on the distribution of NSAID-exposed women (PS fine stratification weight) [33–35]; weights for the NSAID-exposed group are set to 1 and the referent group are reweighted based on the number of treated patients residing within their stratum ([number of exposed in PS stratum i / number of total exposed] / [number of referent in PS stratum i / number of total referent]). We then estimated PS-adjusted relative risks (RRs) with 95% confidence intervals (CIs), or the average treatment effect among the treated, by using generalized linear models (PROC GENMOD with a weight statement, log-link function, and binomial distribution [log-binomial model]); adjustment was achieved through weighting. The primary analysis aimed to address the safety of NSAIDs versus no drug treatment by comparing prenatal NSAID exposure to unexposed women in the etiologically relevant exposure window; this would assess the safety of exposure to NSAIDs. We used SAS Enterprise version 6.1 (SAS Institute, Cary, NC, USA) for all statistical analyses, and this study is reported as per the Strengthening the Reporting of Observational Studies in Epidemiology guideline (**S1 STROBE Checklist**).

#### Secondary analysis

We conducted four secondary analyses, where PS were reestimated for each analysis. First, we compared early prenatal exposure to NSAIDs against (1) early prenatal acetaminophen use, defined as women prescribed ≥2 acetaminophen during the etiologically relevant window (to address the comparative safety of NSAIDs versus a commonly used medication with similar indications to minimize potential confounding by indication); (2) past users of NSAIDs, defined as women exposed to NSAIDs before but not during pregnancy (to address the safety of continuing NSAIDs during pregnancy). Second, given that aspirin was not included as NSAIDs in this study due to discrepancies in medical practice and indications of these drugs (nonaspirin NSAIDs versus aspirin), we also compared early prenatal exposure to aspirin against unexposed pregnancies; an analogous exposure definition to that of the main analysis for NSAIDs was used, but further excluding pregnancies exposed to ≥1 NSAIDs during early pregnancy across both aspirin-exposed and unexposed groups. Third, we repeated the primary analysis but redefined the exposure of interest by the type (nonselective NSAIDs, COX-2 selective inhibitors) and three most frequently prescribed individual NSAIDs. Last, we conducted subgroup analyses for cumulative duration of NSAID use (<5, 5 to 10, >10 days) to address duration–response relations.

#### Sensitivity analysis

Nine distinct sensitivity analyses were done to test the robustness of our main findings, where the overall findings were interpreted in light of these results [36]. First, we restricted the cohort to nulliparous pregnancies to account for correlations among women with multiple pregnancies. Second, we restricted to singleton pregnancies to eliminate the potential confounding effect of multiple gestations. Third, we restricted to pregnant women with data on health examination records to assess residual confounding effects from body mass index and smoking status. Fourth, we restricted to pregnancies with indications for NSAIDs (e.g., inflammatory disease, respiratory infection, fever) to further address confounding by indication. Moreover, we also restricted to pregnancies with severe respiratory infections (i.e., diagnosis of respiratory infections with antibiotic prescriptions or hospital admissions). Alternatively, we also adjusted for the number of (a) outpatient visits, (b) inpatient admissions, and (c) emergency department visits with respiratory infection diagnosis during early pregnancy in the outcome model as continuous variables as proxies of respiratory infection severity. Fifth, for cohort 1 only, we redefined the exposure window as the fourth to 10th week of the gestational period, a previously reported duration of organogenesis. Sixth, we conducted a negative control analysis for all outcomes by redefining the exposure window as the 5 to 8 months before the LMP to examine the possibility of residual confounding in our data. Seventh, we excluded pregnancies exposed to ≥1 NSAIDs any time during pregnancy after the first trimester (cohort 1) or early pregnancy (cohort 2); accordingly, this new definition would allow the specific assessment of exposure to NSAIDs “only” during early pregnancy. Eighth, we performed sibling-matched analyses by using conditional logistic regression models that stratified on the mother’s unique identification number to estimate odds ratios (ORs) for major congenital malformations and low birth weight to address potential confounding from within-family shared factors; only sibling pairs with discordant exposure status contributed to the estimates [37]. Last, we evaluated the potential effect of excluding pregnancies that were terminated through quantitative bias analyses as we included only pregnancies ending in live births (**S1 Appendix**).

## Results

**Fig 1:**
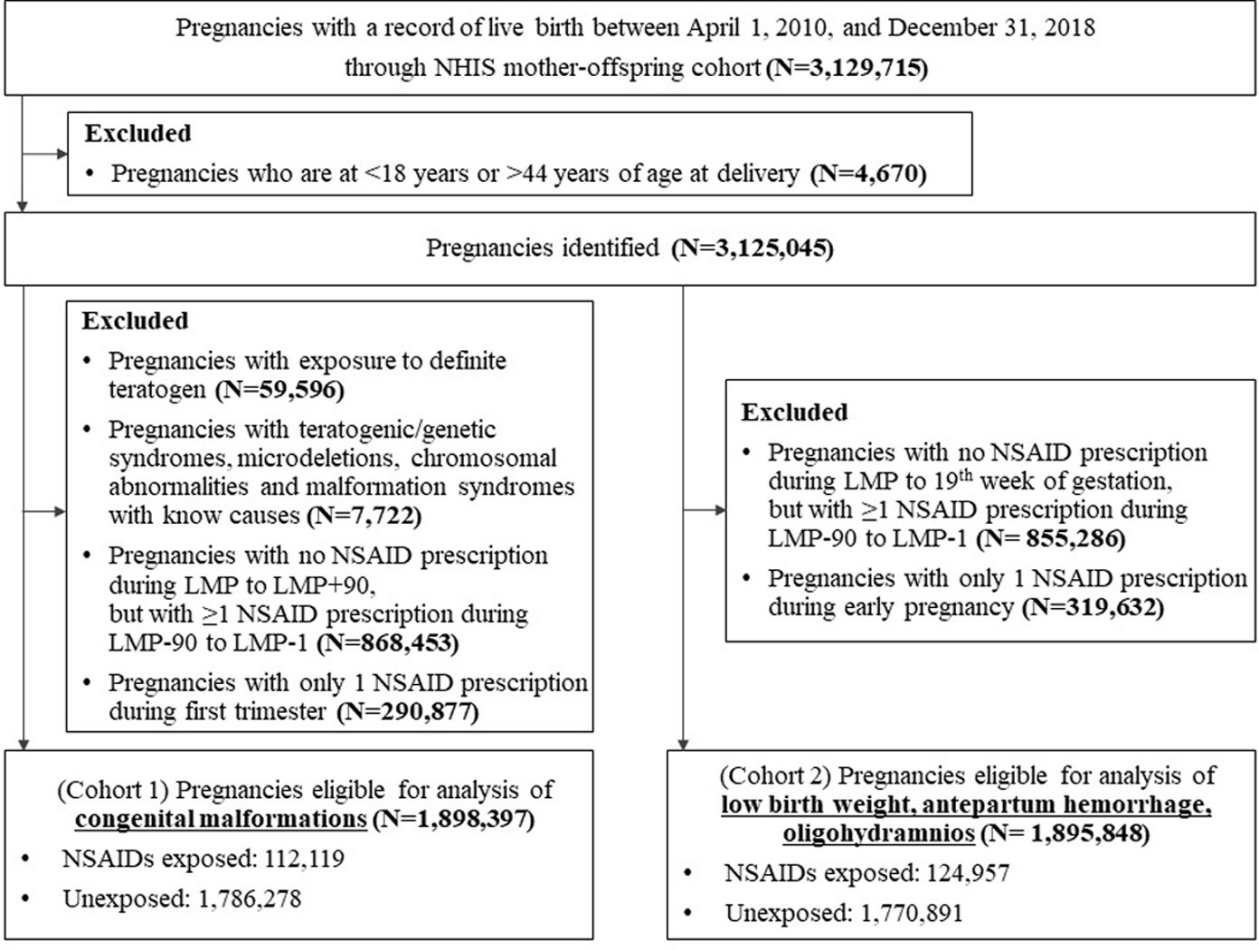
Study flow chart. LMP, last menstrual period; NHIS, National Health Insurance Service; NSAID, nonsteroidal anti-inflammatory drug. *First trimester is defined as the period between the LMP to LMP+90 (cohort 1). ^†^Early pregnancy is defined as the period between LMP to 19 week of gestation (cohort 2).

Of 3,129,715 eligible pregnancies between April 2010 and December 2018, we identified 1,898,397 and 1,895,848 pregnancies for the analyses of congenital malformations (cohort 1) and nonmalformations (cohort 2), respectively (**Fig 1**).

There were 112,119 (5.9%) pregnancies prenatally exposed to NSAIDs in the first trimester (cohort 1) and 124,957 (6.6%) pregnancies prenatally exposed to NSAIDs during early pregnancy (cohort 2). Important imbalances (absolute SMDs ≥0.1) in health insurance type, nulliparity, several comorbid conditions (e.g., asthma, respiratory infection) or co-medication use (e.g., anxiolytics, antiacids), and all general markers of burden of disease were observed between NSAIDs-exposed and unexposed pregnancies before applying PS fine stratification weights. However, after weighting, balance was achieved for all covariates between groups, with absolute SMDs <0.1 (**Table 1**); baseline characteristics for NSAID-exposed versus acetaminophen-exposed pregnancies (active comparator comparison) before and after PS fine stratification weights are shown in **S3 Table**.

**Table 1:** Baseline characteristics of pregnancies exposed to NSAID during the first trimester (cohort 1) or early pregnancy (cohort 2) before and after PS fine stratification weights.

	Cohort 1				Cohort 2			
	NSAID (*n* = 112,119)	Unexposed (*n* = 1,786,278)	SMD^†^		NSAID (*n* = 124,939)	Unexposed (*n* = 1,767,344)	SMD^†^	
			Before PS weight	After PS* weight			Before PS weight	After PS* weight
**Age, years, mean (SD)**	32.1 (4.4)	31.9 (4.2)	0.050	−0.002	32.1 (4.4)	31.9 (4.2)	0.042	0.000
**Age group, years, n (%)**								
18–25	8,248 (7.4)	117,937 (6.6)	0.030	−0.001	9,375 (7.5)	116,674 (6.6)	0.036	−0.001
26–30	29,518 (26.3)	509,865 (28.5)	−0.050	0.002	32,929 (26.4)	505,358 (28.5)	−0.049	0.002
31–35	50,029 (44.6)	826,552 (46.3)	−0.033	0.002	55,722 (44.6)	819,303 (46.3)	−0.034	0.002
36–40	21,445 (19.1)	296,862 (16.6)	0.065	−0.003	23,717 (19)	294,617 (16.6)	0.061	−0.003
41–44	2,879 (2.6)	35,062 (2)	0.041	−0.001	3,214 (2.6)	34,939 (2.0)	0.040	−0.002
**Medical aid recipients, n (%)**	1,990 (1.8)	7,792 (0.4)	**0.128**	0.017	2,265 (1.8)	7,736 (0.4)	**0.131**	0.016
**Income level, n (%)**								
First quartile	24,885 (22.2)	338,308 (18.9)	0.081	0.002	27,753 (22.2)	335,335 (18.9)	0.081	0.002
Second quartile	27,563 (24.6)	432,097 (24.2)	0.009	0.001	30,699 (24.6)	428,468 (24.2)	0.009	0.002
Third quartile	37,256 (33.2)	630,503 (35.3)	−0.044	−0.001	41,463 (33.2)	625,081 (35.3)	−0.045	−0.001
Fourth quartile	22,415 (20)	385,370 (21.6)	−0.039	−0.003	25,042 (20.0)	382,007 (21.6)	−0.038	−0.003
**Region, n (%)**								
Metropolitan	75,888 (67.7)	1,259,308 (70.5)	−0.061	−0.005	85,188 (68.2)	1,247,335 (70.4)	−0.049	−0.005
Urban	36,227 (32.3)	525,558 (29.4)	0.063	0.005	39,764 (31.8)	522,151 (29.5)	0.051	0.005
Rural	‡	1,412 (0.1)	−0.037	−0.002	5 (0.0)	1,405 (0.1)	−0.037	−0.002
**Obstetric characteristics, n (%)**								
Nulliparity	38,975 (34.8)	976,378 (54.7)	**−0.408**	−0.012	43,836 (35.1)	969,391 (54.7)	**−0.403**	−0.011
Multiple gestation	2,229 (2)	31,137 (1.7)	0.018	−0.003	2,468 (2.0)	30,885 (1.7)	0.017	−0.004
**Comorbid conditions, n (%)**								
Anxiety	2,252 (2)	12,032 (0.7)	**0.116**	0.001	2,595 (2.1)	12,299 (0.7)	**0.118**	0.001
Asthma	7,997 (7.1)	35,396 (2)	**0.249**	0.002	9,197 (7.4)	36,730 (2.1)	**0.251**	0.002
Depression	1,864 (1.7)	9,912 (0.6)	**0.106**	−0.003	2,186 (1.7)	10,286 (0.6)	**0.109**	−0.003
Type 1 and 2 diabetes	1,446 (1.3)	9,430 (0.5)	0.080	0.001	1,722 (1.4)	10,279 (0.6)	0.081	0.000
Epilepsy/seizures	190 (0.2)	1,758 (0.1)	0.019	0.001	365 (0.3)	2,770 (0.2)	0.029	0.001
Gastrointestinal diseases	68,033 (60.7)	471,161 (26.4)	**0.737**	0.003	76,737 (61.4)	482,890 (27.3)	**0.732**	0.003
Hypertension	1,444 (1.3)	11,741 (0.7)	0.064	0.003	1,925 (1.5)	14,778 (0.8)	0.065	0.002
Renal disease	538 (0.5)	4,441 (0.2)	0.038	−0.001	635 (0.5)	4,739 (0.3)	0.039	−0.002
Thyroid disorders	10,227 (9.1)	158,999 (8.9)	0.008	0.000	13,791 (11.0)	185,457 (10.5)	0.018	−0.001
Alcohol or drug dependence	150 (0.1)	890 (0)	0.028	−0.001	195 (0.2)	905 (0.1)	0.033	0.000
Tobacco dependence	‡	34 (0)	0.000	0.000	‡	35 (0.0)	−0.001	0.000
Endometriosis	821 (0.7)	11,511 (0.6)	0.011	0.000	949 (0.8)	11,641 (0.7)	0.012	0.000
Polycystic ovarian syndrome	1,011 (0.9)	17,128 (1)	−0.006	−0.003	1,143 (0.9)	16,974 (1.0)	−0.005	−0.002
Respiratory infection	100,261 (89.4)	732,209 (41)	**1.181**	0.018	113,165 (90.6)	775,201 (43.8)	**1.149**	0.019
Inflammatory diseases	1,249 (1.1)	5,550 (0.3)	0.096	−0.007	1,469 (1.2)	5,658 (0.3)	**0.100**	−0.009
Pain	2,234 (2)	8,507 (0.5)	**0.138**	0.003	2,613 (2.1)	9,117 (0.5)	**0.139**	0.002
Fever	5,479 (4.9)	26,867 (1.5)	**0.193**	0.002	7,068 (5.7)	31,327 (1.8)	**0.207**	0.001
Migraine/headache	12,360 (11)	56,830 (3.2)	**0.309**	0.005	15,108 (12.1)	64,514 (3.6)	**0.318**	0.004
**Co-medication use, n (%)**								
Antibiotics	105,079 (93.7)	881,593 (49.4)	**1.129**	0.013	117,637 (94.1)	930,170 (52.5)	**1.067**	0.016
Antiepileptics	1,475 (1.3)	5,584 (0.3)	**0.112**	−0.002	1,992 (1.6)	6,890 (0.4)	**0.122**	−0.003
Antidepressants	4,138 (3.7)	19,047 (1.1)	**0.173**	−0.001	4,734 (3.8)	19,199 (1.1)	**0.176**	−0.001
Antihypertensives	3,959 (3.5)	20,904 (1.2)	**0.156**	0.002	4,637 (3.7)	22,333 (1.3)	**0.158**	0.001
Antipsychotics	596 (0.5)	3,593 (0.2)	0.055	−0.002	741 (0.6)	3,853 (0.2)	0.059	−0.004
Anxiolytics	26,796 (23.9)	167,564 (9.4)	**0.397**	0.003	30,124 (24.1)	168,350 (9.5)	**0.398**	0.003
Azoles	34,103 (30.4)	368,080 (20.6)	**0.226**	−0.004	41,697 (33.4)	405,549 (22.9)	**0.234**	−0.002
Thyroid hormones	3,527 (3.1)	57,762 (3.2)	−0.005	−0.001	4,361 (3.5)	62,401 (3.5)	−0.002	−0.001
Fertility drugs	5,540 (4.9)	103,650 (5.8)	−0.038	−0.004	6,435 (5.1)	103,216 (5.8)	−0.030	−0.005
Hypnotics	2,307 (2.1)	8,616 (0.5)	**0.141**	0.004	2,649 (2.1)	8,739 (0.5)	**0.144**	0.004
Insulin	756 (0.7)	4,680 (0.3)	0.060	0.000	965 (0.8)	5,257 (0.3)	0.065	0.000
Non-insulin antidiabetic drugs	712 (0.6)	4,326 (0.2)	0.059	0.000	797 (0.6)	4,302 (0.2)	0.060	−0.001
Lipid-lowering drug	717 (0.6)	3,257 (0.2)	0.072	0.004	819 (0.7)	3,238 (0.2)	0.073	0.004
Opioid analgesics	82,970 (74)	457,345 (25.6)	**1.106**	0.012	94,144 (75.3)	487,149 (27.5)	**1.090**	0.013
Antiacid	87,517 (78.1)	604,580 (33.8)	**0.995**	0.007	97,696 (78.2)	613,176 (34.6)	**0.978**	0.008
Corticosteroids	64,171 (57.2)	429,873 (24.1)	**0.717**	0.006	72,319 (57.9)	444,971 (25.1)	**0.705**	0.006
Triptans	747 (0.7)	2,248 (0.1)	0.086	0.006	883 (0.7)	2,254 (0.1)	0.090	0.005
Antiemetics	24,318 (21.7)	195,184 (10.9)	**0.294**	0.008	29,928 (24)	212,566 (12)	**0.315**	0.007
Medication for asthma/COPD	33,731 (30.1)	151,875 (8.5)	**0.569**	0.009	38,259 (30.6)	157,616 (8.9)	**0.567**	0.009
DMARD	1,374 (1.2)	12,232 (0.7)	0.056	−0.002	1,587 (1.3)	12,184 (0.7)	0.059	−0.001
Immunosuppressants	6 (0)	93 (0)	0.000	0.000	6 (0)	91 (0)	0.000	0.000
**General markers of burden of illness**								
OCI, mean (SD)	0.8 (1)	0.6 (0.9)	**0.265**	−0.003	0.8 (1.0)	0.6 (0.9)	**0.261**	−0.004
No. of distinct diagnoses, mean (SD)	7.3 (4.8)	3.5 (3.2)	**0.939**	0.030	7.3 (4.8)	3.5 (3.2)	**0.942**	0.031
No. of distinct prescription drugs, excluding NSAIDs, mean (SD)	6.7 (5.1)	2.7 (3.1)	**0.943**	0.035	6.7 (5.1)	2.7 (3.1)	**0.942**	0.039
Emergency room visits, n (%)	10,398 (9.3)	78,990 (4.4)	**0.193**	0.004	11,712 (9.4)	78,120 (4.4)	**0.197**	0.005
Patients hospitalized, n (%)	9,966 (8.9)	79,857 (4.5)	**0.178**	0.005	11,186 (9.0)	79,049 (4.5)	**0.180**	0.006
No. of outpatient visits, mean (SD)	8.8 (8.5)	3.8 (4.4)	**0.741**	0.007	8.8 (8.5)	3.8 (4.4)	**0.741**	0.006
No. of pregnancy-related hospital visits during first month, n (%)	34,383 (30.7)	720,895 (40.4)	**−0.204**	−0.005	38,958 (31.2)	714,348 (40.3)	**−0.192**	−0.005

COPD, chronic obstructive pulmonary disease; DMARD, disease-modifying antirheumatic drugs, NSAID, nonsteroidal anti-inflammatory drug; OCI, obstetric comorbidity index; PS, propensity score; SD, standard deviation; SMD, standardized mean difference.

*To account for PS, the unexposed observations were weighted using the distribution of the exposed observations among 50 PS strata. Observations from the nonoverlapping regions of the PS distributions were trimmed.

^†^Value >0.1 indicates significant imbalance between the exposed and unexposed groups.

^‡^Counts less than five are suppressed due to internal regulations of the National Health Insurance Service, South Korea.

**Fig 2:**
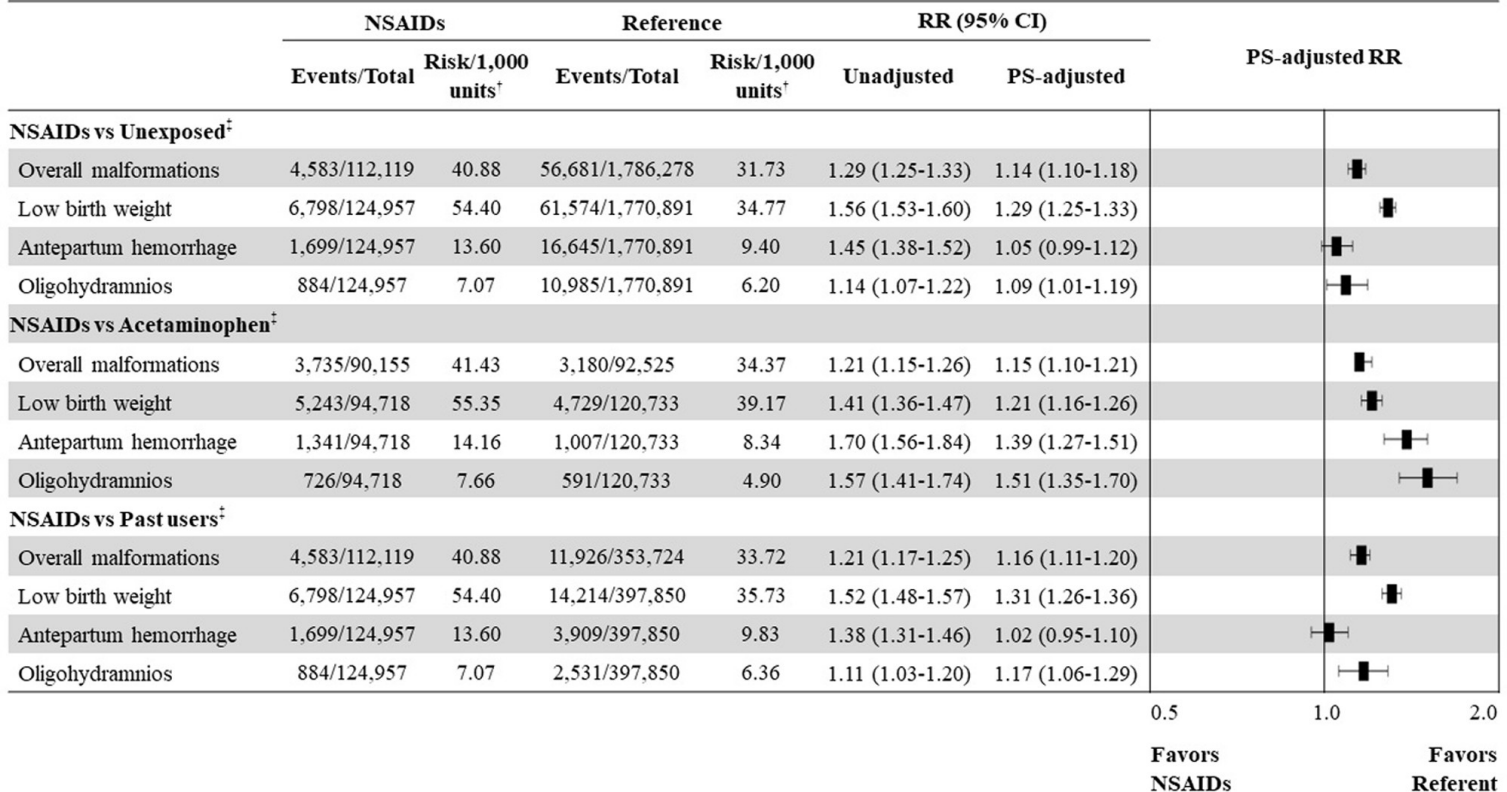
Risk of neonatal and maternal adverse outcomes associated with NSAID exposure during the first trimester or early pregnancy compared with three distinct referent groups. CI, confidential interval; NSAID, nonsteroidal anti-inflammatory drug; PS, propensity score; RD, risk difference; RR, relative risk. *RD_1,000_ = risk difference per 1,000 births or pregnancies. ^†^Units: births for outcomes of overall congenital malformations and low birth weights; pregnancies for outcomes of antepartum hemorrhage and oligohydramnios. ^‡^Associated risk of major congenital malformations and other adverse outcomes in women who used NSAIDs in early pregnancy was compared with three distinct pregnancy groups: women not prescribed any NSAIDs (unexposed), women prescribed ≥2 acetaminophen (acetaminophen), and women who stopped taking NSAIDs during pregnancy but were prescribed them before (past users).

Comparing pregnancies exposed to NSAIDs to those unexposed (primary analysis), the PS-adjusted RRs were 1.14 (95% CI, 1.10 to 1.18) for overall malformations, 1.29 (1.25 to 1.33) for low birth weight, 1.05 (0.99 to 1.12) for antepartum hemorrhage, and 1.09 (1.01 to 1.19) for oligohydramnios. In the results of secondary analyses that compared with acetaminophen-exposed pregnancies or past users of NSAIDs, generally similar trends of associations were observed for the risks of study outcomes (**Fig 2**); results of aspirin-exposed versus unexposed pregnancies were also similar to the primary analysis (**S4 Table**). The risk of heart defects remained significantly higher across all comparisons of NSAID exposure during the first trimester with unexposed pregnancies (PS-adjusted RR, 1.19 [95% CI, 1.13 to 1.24]), acetaminophen-exposed pregnancies (1.17 [1.10 to 1.25]), and past users (1.19 [1.13 to 1.25]) (**S5, S6, and S7 Tables**).

**Fig 3:**
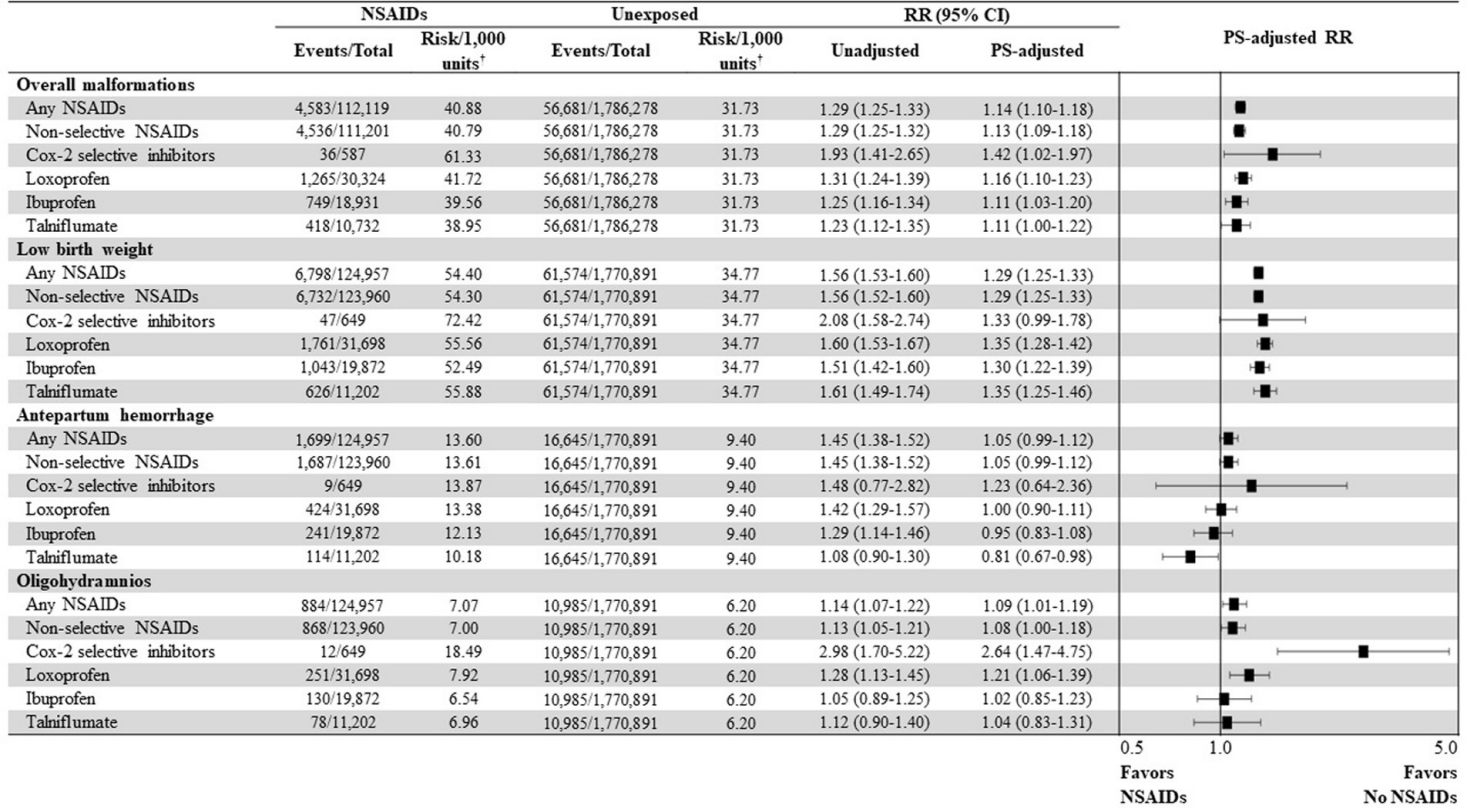
Risk of neonatal and maternal adverse outcomes associated with prenatal NSAID exposure during early pregnancy, depending on the type of NSAIDs and the three most frequently prescribed individual NSAIDs. CI, confidential interval; COX, cyclooxygenase; NSAID, nonsteroidal anti-inflammatory drug; PS, propensity score; RR, relative risk. *Units: births for outcomes of overall congenital malformations and low birth weights; pregnancies for outcomes of antepartum hemorrhage and oligohydramnios.

Results of secondary analyses that assessed the type of or frequently used individual NSAIDs were also largely consistent with the primary analysis; results for COX-2 selective inhibitors had wide CIs likely due to small number of events and pregnancies. Of note, the risk of oligohydramnios was more than 2-fold higher with COX-2 selective inhibitors versus unexposed pregnancies (PS-adjusted RR, 2.64 [95% CI, 1.47 to 4.75]) (**Fig 3**). Meanwhile, a clear duration–response relation was observed only for low birth weight as the PS-adjusted RRs increased proportionally with cumulative duration of NSAID use in early pregnancy (<5 days: PS-adjusted RR, 1.22 [95% CI, 1.17 to 1.28]; 5 to 10 days: 1.28 [1.23 to 1.38]; >10 days: 1.48 [1.39 to 1.58]); the risks of overall malformations (1.23 [1.14 to 1.33]) and low birth weight (1.48 [1.39 to 1.58]) were highest when using NSAIDs for >10 days during early pregnancy (**Table 2**).

**Table 2:** Risks of neonatal and maternal adverse outcomes associated with prenatal NSAID exposure during early pregnancy, stratified on the cumulative duration of NSAID use.

	NSAIDs		Unexposed		Relative Risk (95% CI)	
Subgroups	No. of Event/total	Risk/1,000 units*	No. of Event/total	Risk/1,000 units*	Unadjusted	PS-adjusted
**Overall congenital malformations** ^†^						
**Cumulative duration**						
<5 days	1,490/37,542	39.69	56,681/1,786,278	31.73	1.25 (1.19–1.32)	1.13 (1.08–1.20)
5–10 days	2,251/57,225	39.34	56,681/1,786,278	31.73	1.24 (1.19–1.29)	1.11 (1.06–1.16)
>10 days	842/17,352	48.52	56,681/1,786,278	31.73	1.53 (1.43–1.63)	1.23 (1.14–1.33)
**Low birth weight** ^†^						
**Cumulative duration**						
<5 days	2,190/44,219	49.53	61,574/1,770,891	34.50	1.42 (1.37–1.48)	1.22 (1.17–1.28)
5–10 days	3,225/61,161	52.73	61,574/1,770,891	34.50	1.52 (1.47–1.57)	1.28 (1.23–1.33)
>10 days	1,383/19,577	70.64	61,574/1,770,891	34.50	2.03 (1.93–2.14)	1.48 (1.39–1.58)
**Antepartum hemorrhage** ^†^						
**Cumulative duration**						
<5 days	576/44,219	13.03	16,645/1,770,891	9.40	1.39 (1.28–1.51)	1.08 (0.99–1.18)
5–10 days	834/61,161	13.64	16,645/1,770,891	9.40	1.45 (1.35–1.55)	1.05 (0.98–1.14)
>10 days	289/19,577	14.76	16,645/1,770,891	9.40	1.57 (1.40–1.76)	1.03 (0.90–1.18)
**Oligohydramnios** ^†^						
**Cumulative duration**						
<5 days	304/44,219	6.87	10,985/1,770,891	6.20	1.11 (0.99–1.24)	1.08 (0.96–1.22)
5–10 days	420/61,161	6.87	10,985/1,770,891	6.20	1.11 (1.00–1.22)	1.07 (0.96–1.19)
>10 days	160/19,577	8.17	10,985/1,770,891	6.20	1.32 (1.13–1.54)	1.19 (1.00–1.42)

CI, confidential interval; NSAID, nonsteroidal anti-inflammatory drug; PS, propensity score; RR, relative risk.

*Units: births for outcomes of overall congenital malformations and low birth weights; pregnancies for outcomes of antepartum hemorrhage and oligohydramnios.

^†^Mean (standard deviation) and median (Q1, Q3) cumulative duration for each outcome were: 8.6 (6.5) and 7.0 (5.0, 10.0) for overall congenital malformations; 8.8 (8.1) and 7.0 (5.0, 10.0) for low birth weight, antepartum hemorrhage, and oligohydramnios.

**Fig 4:**
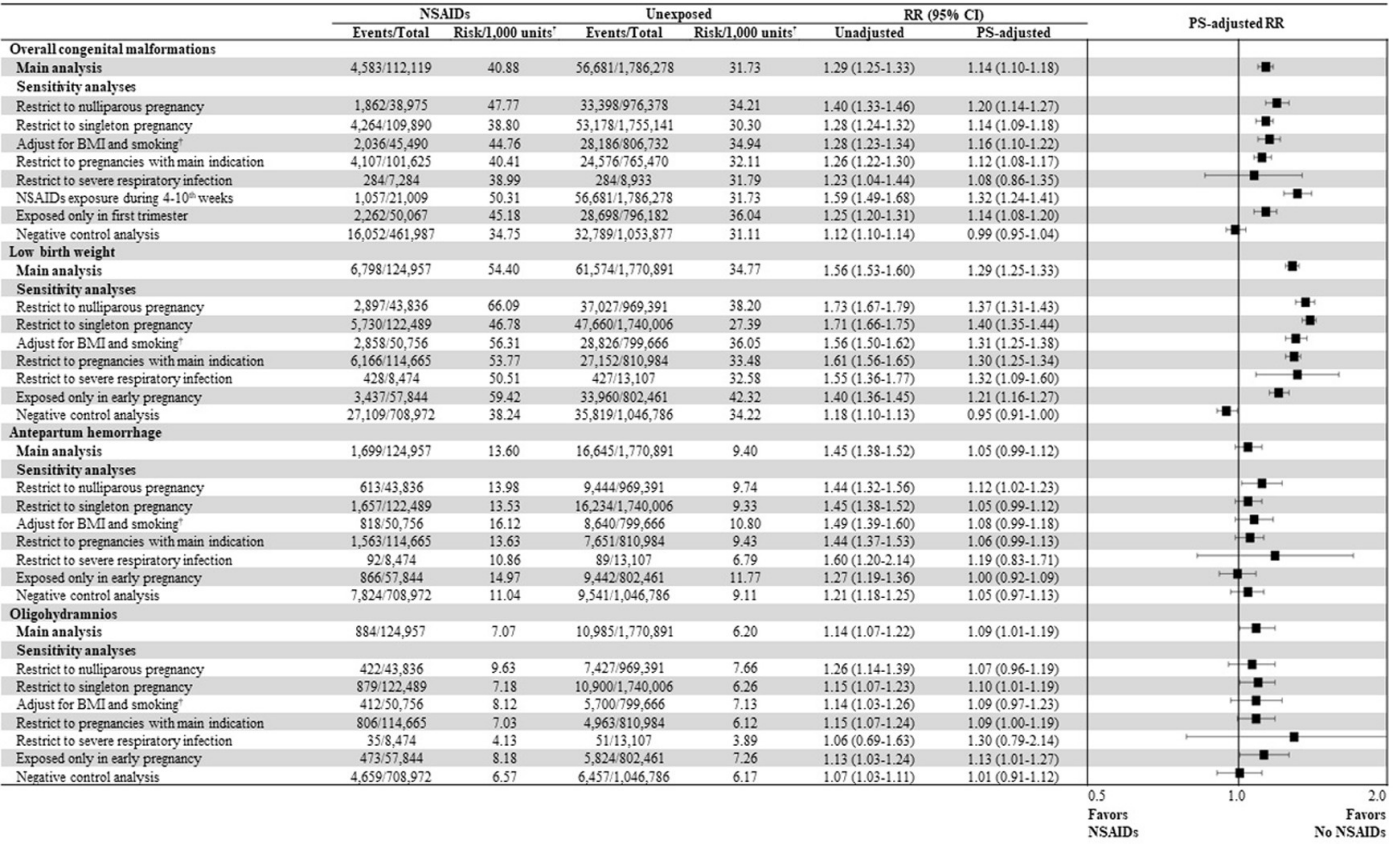
Results of sensitivity analyses for the risks of neonatal and maternal adverse outcomes associated with prenatal NSAID exposure during early pregnancy. CI, confidential interval; NSAID, nonsteroidal anti-inflammatory drug; PS, propensity score; RR, relative risk. ^†^Restrict the study cohort to those who underwent health screening examination for whom information on BMI and smoking was available. *Units: births for outcomes of overall congenital malformations and low birth weights; pregnancies for outcomes of antepartum hemorrhage and oligohydramnios.

Our main findings were generally consistent across all sensitivity analyses, with regard to the point estimates (**Fig 4 and S8, S9, S10 and S11 Tables**). Risks of overall malformations (OR, 1.13 [95% CI, 1.00 to 1.29]) and low birth weight (1.30 [1.14 to 1.49]) associated with early prenatal exposure to NSAIDs versus unexposed pregnancies remained statistically significant in sibling-matched analyses that further accounted for familial factors (**S12 Table**). Results of the quantitative bias analysis found that the corrected RR for overall malformations and congenital heart defects remained <1.41 under the most extreme scenario (**S1 Appendix and S2 and S3 Figs**).

## Discussion

### Main findings

This large-scale, nationwide cohort study of 1.8 million pregnancies found that, as compared with unexposed pregnancies, early prenatal exposure to NSAIDs was significantly associated with higher risks for most outcomes examined, especially for major congenital malformations, likely driven by heart defects, low birth weight, and oligohydramnios. In particular, the associations for overall malformations, low birth weight, and oligohydramnios remained significant despite comparing NSAIDs against acetaminophen or past users. Moreover, associations of adverse neonatal and maternal outcomes may be stronger with COX-2 selective inhibitors or use of NSAIDs for >10 days. Generally consistent findings, with regard to point estimates, observed across a range of sensitivity analyses provide some reassurance; however, residual confounding by indication or from unmeasured confounders may still be present due to inherent limitations of the observational nature of this study using routine care data.

### Comparison with previous studies

The observed positive associations of overall malformations and low birth weight with early prenatal NSAID use were inconsistent with existing literature (**S1 Table**), which generally reported null associations [10,16–18,20,38–44]. However, previous studies had important limitations, making it difficult to make any meaningful comparisons. For example, most of previously reported effect estimates had very wide CIs, likely due to small sample size, resulting in uncertainty in the study’s ability to examine the research question with accuracy and precision. Moreover, former studies were unable to fully account for various sources of confounding bias (e.g., confounding by indication) by comparing against only unexposed pregnancies. Furthermore, evidence of this topic that addressed the effects of NSAIDs on Asians was absent to our knowledge. Meanwhile, this study accounted for these limitations by not only accounting for >50 covariates but also comparing NSAIDs to unexposed as well as acetaminophen and past users among 1.8 million pregnancies in South Korea. Hence, we believe that our findings provide evidence from a real-world setting for healthcare providers on the safety of NSAIDs during early pregnancy in an Asian pregnant population, which could help fill current knowledge gaps on this space of both clinical and public health importance.

Unlike congenital malformations and low birth weight, there were little to no previous studies for comparison on antepartum hemorrhage or oligohydramnios with nonselective NSAIDs or COX-2 inhibitor use in early pregnancy. With future studies, preferably randomized clinical trials, warranted to corroborate our findings, the observed risk of antepartum hemorrhage and oligohydramnios associated with early prenatal NSAID use versus acetaminophen-exposed pregnancies suggest cautious use of NSAIDs in this period, as well as the possibility that acetaminophen could be the safer option. While few mechanisms of action of acetaminophen different to that of NSAIDs, such as indirect inhibition of COX, could possibly explain this observation [45–49], a more in-depth investigation is needed. Moreover, given that oligohydramnios can be an end point of multiple pathways, including fetal kidney impairment, intrauterine growth restriction, and rupture of the fetal membranes, and the observational nature of this study being able to only examine an association and not causation, cautious interpretation is warranted. Thus, based on these findings and the fact that acetaminophen has relatively minimal anti-inflammatory effects versus NSAIDs [49], the decision to prescribe or take NSAIDs over acetaminophen during pregnancy should be carefully considered only for the treatment of inflammatory conditions.

Given the large size of this study, we assessed with higher precision the association of neonatal and maternal adverse outcomes following prenatal exposure to COX-2 inhibitors than former studies [20,42]. While one study found a similar estimate for major congenital malformations with COX-2 inhibitor use in the first trimester (RR, 1.40 [95% CI, 0.70 to 2.78]) [42] to our study (PS-adjusted RR, 1.42 [95% CI, 1.02 to 1.97]), we found statistically significant associations by having greater power; another prior study also had very wide CIs (0.96 [0.28 to 3.26]) [20]. As studies on this topic were also lacking for comparisons, further investigations using multiple data sources to overcome sample size issues are likely needed to accurately examine the safety of COX-2 inhibitors in early pregnancy.

### Strengths and limitations

To our knowledge, this is the largest observational study that examined the association between early prenatal NSAID use and the risk of neonatal and maternal adverse outcomes by using nationwide data of more than 3 million mother–offspring pairs augmented with advanced epidemiological and statistical methods. Notably, this is the first study, to our knowledge, that assessed the risk of several outcomes, including antepartum hemorrhage and oligohydramnios, with NSAID use in an Asian population. The large sample allowed for analyses of rare outcomes, COX-2 inhibitors, and duration–response relations with relatively high precision. Moreover, exposure to NSAIDs is likely to be unaffected by recall bias given the use of routinely collected electronic prescribing records to define exposure status. Furthermore, largely consistent findings with regard to point estimates across all sensitivity analyses support the robustness of our primary findings. Finally, the deterministic mother–offspring link based on algorithms developed, validated, and provided for research purposes by the NHIS further improved the study’s accuracy and reliability.

This study also has a few limitations, which should be carefully taken into account when interpreting our findings. First, exposure misclassification is possible as NSAIDs may be purchased over-the-counter, which are unavailable for assessment in the claims data used. Hence, differential misclassification in unexposed pregnancies cannot be ruled out as women obtaining their NSAIDs over-the-counter are unlikely to have been prescribed NSAIDs as well; the true proportion of NSAID-exposed pregnancies could be larger among unexposed pregnancies. Moreover, although we were unable to determine whether patients actually took the NSAIDs prescribed, we applied a strict exposure definition of ≥2 prescriptions of NSAIDs to address for this uncertainty; under reasonable assumptions, if a woman refilled the respective drug’s prescription, she probably took them. Meanwhile, exposure misclassification due to inaccurate timing of gestational age is another possibility, which, however, would be expected to bias the estimates towards the null. Second, we could have underestimated the risk of antepartum hemorrhage or oligohydramnios as local clinicians and specialists do not always report them in routine clinical practice. Yet, this misclassification is likely to be nondifferential between groups as, although failure to record certain conditions are related with demographic factors; these were all well balanced in the weighted cohort. Hence, any bias arising from this, under reasonable assumptions, would have led the estimates towards the null. Third, residual confounding by indication is still possible. However, the results of the analysis that compared NSAIDs with acetaminophen and the point estimates of the sensitivity analysis that restricted to pregnancies with indications of NSAIDs (e.g., inflammatory disease) or severe respiratory infections, and exclusive use in early pregnancy suggest that were consistent with the main findings. Fourth, residual confounding from unmeasured confounders cannot be ruled out despite rigorous adjustment of 52 potential confounders and the sibling-matched analysis that further accounted for familial factors. Fifth, assessment of first trimester or early pregnancy exposure starting from the LMP adds 2 weeks of exposure prior to conception, which, with the short NSAID half-life, would again bias toward the null. Sixth, we restricted our study cohorts to live births only, which could have led to selection bias given that severe malformations resulting in pregnancy terminations would have been missed. However, the results of quantitative bias analyses showed that the corrected RR for overall congenital malformations and congenital heart defects remained <1.41 under the most extreme scenario. It indicates that selection bias, if present, is likely to have been minimal (**S1 Appendix** and **S2 and S3 Figs**). Last, given the nature of this observational study and the use of routinely collected insurance claims data to examine the safety of NSAIDs in early pregnancy, our findings are limited to suggesting only an association and not causation or causal relationships. Hence, caution is warranted in interpreting our findings.

## Conclusions

In this comprehensive, nationwide cohort study of 1.8 million pregnancies, exposure to NSAIDs during early pregnancy, as compared with no exposure, was associated with slightly higher risks of major congenital malformations, largely driven by heart defects, low birth weight, and oligohydramnios. Notably, neonatal and maternal adverse outcomes were more strongly associated with the use of COX-2 inhibitors or NSAIDs for >10 days during early pregnancy. These findings therefore suggest that clinicians should carefully weigh the benefits of prescribing NSAIDs in early pregnancy against its modest, but possible, risk of neonatal and maternal outcomes. Further, if possible, both clinicians and pregnant women should consider prescribing or receiving nonselective NSAIDs for <10 days, respectively, with continued careful monitoring for any safety signals. Future studies are warranted to corroborate our findings and to examine the causation on this topic.

## Supporting information

S1 STROBE Checklist STROBE, Strengthening the Reporting of Observational Studies in Epidemiology. (DOCX)

S1 Table Previous studies on NSAID and the risk of adverse birth outcomes and pregnancy-related complications. (DOCX)

S2 Table Codes used to define exclusion criteria, exposures, outcomes, and covariates. (DOCX)

S3 Table Baseline characteristics of pregnancies exposed to NSAID versus acetaminophen during the first trimester (cohort 1) or early pregnancy (cohort 2) before and after PS fine stratification weights. (DOCX)

S4 Table Risk of neonatal and maternal adverse outcomes following early prenatal exposure to aspirin versus unexposed pregnancies. (DOCX)

S5 Table Risk of congenital malformations in infants following maternal exposure to NSAID during the first trimester compared with unexposed pregnancies. (DOCX)

S6 Table Risk of congenital malformations in infants following maternal exposure to NSAID during the first trimester compared with acetaminophen-exposed pregnancies. (DOCX)

S7 Table Risk of congenital malformations in infants following maternal exposure to NSAID during the first trimester compared with NSAID past users. (DOCX)

S8 Table Risk of neonatal and maternal adverse outcomes following exposure to NSAIDs during early pregnancy that restricted to pregnancies with severe respiratory infections. (DOCX)

S9 Table Risk of neonatal and maternal adverse outcomes following exposure to NSAIDs during early pregnancy that adjusted for severe respiratory infections. (DOCX)

S10 Table Risk of neonatal and maternal adverse outcomes following exposure to NSAIDs only in first trimester or early pregnancy. (DOCX)

S11 Table Risk of neonatal and maternal adverse outcomes following exposure to NSAIDs only in first trimester or early pregnancy: Subgroup analysis according to cumulative duration of NSAIDs. (DOCX)

S12 Table Risk of congenital malformations and low birth weight in infants following maternal exposure to NSAID during the first trimester according to three distinct referent group: Sibling analyses. (DOCX)

S1 Fig Propensity score distribution of NSAIDs-exposed and referent groups before and after propensity score based fine stratification. (DOCX)

S2 Fig Corrected relative risk for the association between NSAID exposure during the first trimester and overall congenital malformations starting from an observed RR of 1.14. (DOCX)

S3 Fig Corrected relative risk for the association between NSAID exposure during the first trimester and congenital heart defects starting from an observed RR of 1.19. (DOCX)

S1 Appendix Potential effect of including live births only. (DOCX)

S1 Protocol Summary protocol. (DOCX)

## Abbreviations

CI: confidence interval
COX: cyclooxygenase
EUROCAT: European Surveillance of Congenital Anomalies
LMP: last menstrual period
NHIS: National Health Insurance Service
NHIS-NHID: National Health Insurance Service-National Health Insurance Database
NSAIDs: nonsteroidal anti-inflammatory drugs
OR: odds ratio
PS: propensity score
RR: relative risk
SMD: standardized mean difference
