# Supplementary material for: Neonatal and maternal adverse outcomes and exposure to nonsteroidal anti-inflammatory drugs during early pregnancy in South Korea: A nationwide cohort study
Source: PLoS Med. 2023 Feb 27;20(2):e1004183. doi: 10.1371/journal.pmed.1004183 (PMC9970080; doi:10.1371/journal.pmed.1004183)
Supplement: S1 Table — (DOCX) [file pmed.1004183.s002.docx]

**S1 Table.** Previous studies on NSAID and the risk of adverse birth outcomes and pregnancy-related complications.

| **No.** | **Study** | **Data Source** | **Total N (or N controls)** | **Exposure** | **N exposed (or N cases)** | **Outcome** | **RR/OR**  **(95% CI)** | **Comments** |
| --- | --- | --- | --- | --- | --- | --- | --- | --- |
| **Non-congenital malformation outcomes (low birth weight, oligohydramnios, antepartum hemorrhage)** | | | | | | | | |
| 1 | Nielsen, 2001 | Danish birth registry | 18,721 | NSAIDs  (first trimester) | 1,106 | Low birth weight | 0.79 (0.45-1.38) | -Potential confounding by indication and by familial factors |
| 2 | Nezvalová-Henriksen, 2013 | Norwegian Mother and Child Cohort Study and Medical Birth Registry of Norway | 90,417 | Ibuprofen  (first trimester) | 3,034 | Low birth weight | 1.2 (0.9-1.6) | -Potential confounding by indication and by familial factors |
| **Neonatal outcomes (congenital malformations)** | | | | | | | | |
| 1 | Ericson, 2001 | Birth Congenital Malformations Child Cardiology Registry | Not specified | NSAIDs  (first trimester) | Not specified | Any congenital malformation | 1.04 (0.84-1.29) | -Potential confounding by indication and by familial factors  -Information on sample size unavailable |
| 2 | Nielsen, 2001 | Danish birth registry | 18,721 | NSAIDs  (first trimester) | 1,106 | Congenital abnormalities | 1.27 (0.93-1.75) | -Potential confounding by indication and by familial factors |
| 3 | Källén, 2003 | Swedish health registers | 582,745 | NSAIDs  (first trimester) | 7,968 | Cardiovascular defect | 1.24 (0.99-1.55) | -Potential confounding by indication and by familial factors |
| 4 | Cleves, 2004 | US National Birth Defects Prevention Study | 860 | NSAIDs  (first trimester) | 158 | All muscular ventricular septal defects | 1.00 (0.64-1.59) | -Potential confounding by indication and by familial factors  -Small sample size |
| 5 | Ofori, 2006 | Administrative databases of Quebec | 36,387 | NSAIDs  (first trimester) | 1,056 | All congenital anomalies | 2.21 (1.72-2.85) | -Potential confounding by indication and by familial factors |
| 6 | van Gelder, 2011 | Norwegian Mother and Child Cohort Study | 69,929 | NSAIDs  (first trimester) | 3,023 | All selected birth defects | 0.7 (0.4-1.1) | -Potential confounding by indication and by familial factors |
|  |  |  |  |  |  | Congenital heart defects | 0.9 (0.5-1.4) |  |
| 7 | Daniel, 2012 | Israel’s Clalit healthcare database | 110,783 | Nonselective NSAIDs  (first trimester) | 5,153 | Major congenital malformations in general | 1.07 (0.96-1.21) | -Potential confounding by indication and by familial factors |
|  |  |  |  | COX-2 inhibitors  (first trimester) | 114 |  | 1.40 (0.70-2.78) |  |
| 8 | Nezvalová-Henriksen, 2013 | Norwegian Mother and Child Cohort Study and Medical Birth Registry of Norway | 90,417 | Ibuprofen  (first trimester) | 3,034 | Any congenital malformation | 1.0 (0.8-1.1) | -Potential confounding by indication and by familial factors |
|  |  |  |  |  |  | Major congenital malformation | 0.9 (0.7-1.2) |  |
| 9 | Cantarutti, 2018 | Italy’s Lombardy healthcare database | 353,081 | Nimesulide  (first trimester) | 627 | Congenital malformations overall | 1.21 (0.90-1.62) | -Potential bias from reverse causality  -Potential confounding by indication and by familial factors |
|  |  |  |  |  |  | Circulatory system anomalies | 1.25 (0.77-2.03) |  |
| 10 | Dathe, 2018 | German Embryotox Pharmacovigilance Institute database | 695 | COX-2 inhibitors  (first trimester) | 139 | Major birth defects | 0.96 (0.28-3.26) | -Potential confounding by indication and by familial factors  -Small sample size |
| 11 | Padberg, 2018 | German Embryotox Pharmacovigilance Institute database | 1,038 | Diclofenac  (first trimester) | 260 | Major birth defects | 0.59 (0.17-2.08) | -Potential confounding by indication and by familial factors  -Small sample size |

**Abbreviation:** NSAID=Non-Steroidal Anti-Inflammatory Drug, OR=Odds Ratio, RR=Relative Risk, CI=Confidence Interval

**References**

1. Ericson A, Källén BA. Nonsteroidal anti-inflammatory drugs in early pregnancy. Reprod Toxicol. 2001;15(4):371-5.

2. Nezvalová-Henriksen K, Spigset O, Nordeng H. Effects of ibuprofen, diclofenac, naproxen, and piroxicam on the course of pregnancy and pregnancy outcome: a prospective cohort study. *Bjog.* 2013;120(8):948-59.

3. Nielsen GL, Sørensen HT, Larsen H, Pedersen L. Risk of adverse birth outcome and miscarriage in pregnant users of non-steroidal anti-inflammatory drugs: population based observational study and case-control study. *Bmj.* 2001;322(7281):266-70.

4. van Gelder MM, Roeleveld N, Nordeng H. Exposure to non-steroidal anti-inflammatory drugs during pregnancy and the risk of selected birth defects: a prospective cohort study. *PLoS One.* 2011;6(7):e22174.

5. Dathe K, Padberg S, Hultzsch S, Köhler LM, Meixner K, Fietz AK, et al. Exposure to cox-2 inhibitors (coxibs) during the first trimester and pregnancy outcome: a prospective observational cohort study. *Eur J Clin Pharmacol.* 2018;74(4):489-95.

6. Källén BA, Otterblad Olausson P. Maternal drug use in early pregnancy and infant cardiovascular defect. *Reprod Toxicol.* 2003;17(3):255-61.

7. Cleves MA, Savell VH, Jr., Raj S, Zhao W, Correa A, Werler MM, et al. Maternal use of acetaminophen and nonsteroidal anti-inflammatory drugs (NSAIDs), and muscular ventricular septal defects. *Birth Defects Res A Clin Mol Teratol.* 2004;70(3):107-13.

8. Ofori B, Oraichi D, Blais L, Rey E, Bérard A. Risk of congenital anomalies in pregnant users of non-steroidal anti-inflammatory drugs: A nested case-control study. *Birth Defects Res B Dev Reprod Toxicol.* 2006;77(4):268-79.

9. Daniel S, Matok I, Gorodischer R, Koren G, Uziel E, Wiznitzer A, et al. Major malformations following exposure to nonsteroidal antiinflammatory drugs during the first trimester of pregnancy. *J Rheumatol.* 2012;39(11):2163-9.

10. Cantarutti A, Franchi M, Rea F, Merlino L, Corrao G. Use of Nimesulide During Early Pregnancy and the Risk of Congenital Malformations: A Population-Based Study from Italy. *Adv Ther.* 2018;35(7):981-92.

11. Padberg S, Tissen-Diabaté T, Dathe K, Hultzsch S, Meixner K, Linsenmeier V, et al. Safety of diclofenac use during early pregnancy: A prospective observational cohort study. *Reprod Toxicol.* 2018;77:122-
