# Supplementary material for: Neonatal and maternal adverse outcomes and exposure to nonsteroidal anti-inflammatory drugs during early pregnancy in South Korea: A nationwide cohort study
Source: PLoS Med. 2023 Feb 27;20(2):e1004183. doi: 10.1371/journal.pmed.1004183 (PMC9970080; doi:10.1371/journal.pmed.1004183)
Supplement: S2 Table — (DOCX) [file pmed.1004183.s003.docx]

**S2 Table**. Codes used to define exclusion criteria, exposures, outcomes, and covariates

| **Categories** | **Codes** |
| --- | --- |
| **Exclusion criteria** | **ICD-10 or ATC codes** |
| Teratogenic/genetic syndromes, microdeletions, chromosomal abnormalities and malformation syndromes with known causes | D821, P350-P351, P371, Q447, Q619, Q751, Q754, Q771-Q772, Q780, Q796, Q85-Q87, Q90-Q93, Q95-Q99 |
| Known or potential teratogens | Antineoplastic agent (L01), warfarin (B01AA03), lithium (N05AN), systemic retinoids (D10BA, D05BB), misoprostol (A02BB01, G02AD06, M01AE56), thalidomide (L04AX02, L04AX04, L04AX06), androgens (G03B, G03E, G03XA), valproic acid (N03AG01), topiramate (N03AX11), carbamazepine (N03AF01), oxcarbazepine (N03AF02), phenobarbital (N03AA02), phenytoin (N03AB02) |
| **Exposures** | **ATC codes** |
| NSAIDs | M01A |
| **Outcomes of interest*** | **ICD-10 codes** |
| Major congenital malformations |  |
| Nervous system | Q00-Q07 |
| Eye | Q100, Q104, Q106-Q109, Q11-Q12, Q130-Q134, Q136-Q139, Q14-Q15 |
| Ear, face, and neck | Q16, Q176-Q178, Q183, Q188 |
| Heart defects | Defects of cardiac chambers and their connections: Q20  Septal defects: Q21 (Ventricular (VSD): Q210, atrial (ASD): Q211, atrioventricular (AVSD): Q212, Tetralogy of Fallot: Q213) Defects of pulmonary and tricuspid valves: Q22  Defects of mitral and aortic valves: Q23  Other cardiac defects: Q240-Q245, Q248-249  Defects of great arteries: Q25  Defects of the great veins: Q260, Q262-Q269 |
| Respiratory system | Q300, Q321, Q323-Q329, Q330, Q332-Q335, Q337-Q339, Q34 |
| Oral clefts | Cleft palate: Q351, Q353, Q355, Q359 Cleft lip with or without cleft palate: Q36-Q37 |
| Digestive system | Q380, Q383-Q389, Q39, Q402-Q409, Q41-Q42, Q431-Q439, Q440-Q443, Q445-Q447, Q45, Q790 |
| Abdominal wall defects | Q792-Q793, Q795 |
| Urinary system | Q60, Q611-Q619, Q620-Q626, Q628-Q629, Q630-Q632, Q634-Q639, Q64, Q794 |
| Genital organs | Q500, Q503, Q504, Q506, Q51, Q520-Q522, Q524, Q526, Q528-Q529, Q540-Q543, Q548-Q549, Q55-Q56 |
| Limb | Q650-Q652, Q658-Q659, Q660, Q679, Q681-Q682, Q686-Q689, Q69, Q70-Q74 |
| Other congenital malformations | Q750, Q77, Q782-Q788, Q80-Q81, Q820-Q824, Q826-Q829, Q860, Q890, Q893-Q894 |
| Low birth weight | P07 |
| Antepartum hemorrhage | O44.1, O46 (from week 20 of gestation to the delivery date) |
| Oligohydramnios | O41.0 (from week 20 of gestation to the delivery date) |
| **Comorbid medical conditions** | **ICD-10 codes** |
| Anxiety | F40-F41 |
| Asthma | J45-J46 |
| Depression | F32-F33 |
| Type 1 and 2 Diabetes | E10-E14 |
| Epilepsy/seizures | G40-G41 |
| Gastrointestinal diseases | K20-K21, K25-K29, K58 |
| Hypertension | I10-I15, O10-O16 |
| Renal disease | E112, E132, E142, I12-I13, N00-N08, N17-N19, N25-N27 |
| Thyroid disorders | E01-E03, E05-E07 |
| Alcohol or drug dependence | F10-F16, F18-F19, Z71.4, Z71.5, Z72.1, Z72.2 |
| Tobacco dependence | F17, Z716, Z720 |
| Endometriosis | N80 |
| Polycystic ovarian syndrome | E282 |
| Respiratory infection | J00-J06, J13-J18, J20- J22, J32, J36, J39.0, J39.1, J40, J69, J85-86 |
| Inflammatory diseases (IBD, SLE, RA, AS) | M05-M09, M30-M32, M33-M34, M351, M353, M45, K50-K51 |
| Pain | R52.0, R52.9 |
| Fever | R50 |
| Migraine/headache | G43-G44, R51 |
| **Obstetric conditions** | **NHIS-NHID procedure codes or ICD 10 codes** |
| Nulliparous | R3131, R3133, R3141, R3143, R4351, R4353, R4361, R4517, R4519, R4507, R4509, R5001, RA361, RA311, RA312, RA315, RA316, RA431, RA432 |
| Multifetal pregnancy | R3133, R3138, R3143, R3148, R4353, R4358, R4516, R4,519, R4520, R5001, R5002, RA312, RA314, RA316, RA318, RA432, RA434 |
| Preterm birth | O42, O601, O603 on mothers’ code at delivery date  P072, P073 on infants’ codes between delivery and delivery + 30 days |
| **Concurrent medication use** | **ATC codes** |
| Antibiotics | J01 |
| Antiepileptics | N03A |
| Antidepressants | N06A |
| Antihypertensives | C03A-E, C07, C08C-D, C09A-D |
| Antipsychotics | N05A |
| Anxiolytics | N05B |
| Azoles | J02AC, D01AC |
| Thyroid hormones | H03AA |
| Fertility drugs | G03G |
| Hypnotics | N05CF |
| Insulin | A10A |
| Non-insulin antidiabetic agents | A10B |
| Lipid lowering drug | C10 |
| Opioid analgesics | N02A |
| Antiacid (PPI/H2RA) | A02BC, A02BA |
| Corticosteroids | H02AB |
| Triptans | N02CC |
| Antiemetics | A04A |
| Medication for asthma/COPD | R03, R06 |
| DMARD | L04AA, L04AB |
| Immunosuppressants | L04AC, L04AD, L04AX |

**Abbreviation:** ATC=Anatomical Therapeutic Chemical Classification, ICD-10=International Classification of Diseases 10th revision, NHIS-NHID=National Health Insurance Service-National Health Information Database

*To increase the specificity, the malformations were defined by the presence of ≥2 diagnoses or ≥1 diagnosis with infant death between delivery and delivery+1 year.
