# Supplementary material for: Neonatal and maternal adverse outcomes and exposure to nonsteroidal anti-inflammatory drugs during early pregnancy in South Korea: A nationwide cohort study
Source: PLoS Med. 2023 Feb 27;20(2):e1004183. doi: 10.1371/journal.pmed.1004183 (PMC9970080; doi:10.1371/journal.pmed.1004183)
Supplement: S3 Table — (DOCX) [file pmed.1004183.s004.docx]

**S3 Table.** Baseline characteristics of pregnancies exposed to NSAID versus acetaminophen during the first trimester (cohort 1) or early pregnancy (cohort 2) before and after PS fine stratification weights

|  | **Cohort 1** | | | | **Cohort 2** | | | |
| --- | --- | --- | --- | --- | --- | --- | --- | --- |
|  | **NSAID**  **(n=90,155)** | **Acetaminophen**  **(n=92,525)** | **SMD^†^** | | **NSAID**  **(n=94,718)** | **Acetaminophen**  **(n=120,733)** | **SMD^†^** | |
|  |  |  | **Before**  **PS weight** | **After**  **PS weight** |  |  | **Before**  **PS weight** | **After**  **PS weight** |
| **Age, years, mean (SD)** | 32.1 (4.4) | 31.7 (4.2) | 0.087 | -0.001 | 32.1 (4.4) | 31.6 (4.2) | 0.131 | 0.000 |
| **Age group, years, n (%)** |  |  |  |  |  |  |  |  |
| 18-25 | 6,665 (7.4) | 7,059 (7.6) | -0.009 | 0.000 | 7,113 (7.5) | 9,582 (7.9) | -0.016 | -0.002 |
| 26-30 | 23,642 (26.2) | 26,677 (28.8) | -0.058 | 0.001 | 24,720 (26.1) | 36,278 (30) | -0.088 | 0.001 |
| 31-35 | 40,028 (44.4) | 42,283 (45.7) | -0.026 | 0.000 | 42,011 (44.4) | 55,251 (45.8) | -0.028 | 0.000 |
| 36-40 | 17,496 (19.4) | 14,792 (16) | 0.090 | -0.001 | 18,413 (19.4) | 17,769 (14.7) | 0.126 | -0.001 |
| 41-44 | 2,324 (2.6) | 1,714 (1.9) | 0.049 | -0.001 | 2,461 (2.6) | 1,853 (1.5) | 0.075 | 0.001 |
| **Medical aid recipients, n (%)** | 1,443 (1.6) | 1,318 (1.4) | 0.014 | -0.005 | 1,521 (1.6) | 1,246 (1) | 0.050 | -0.003 |
| **Income level, n (%)** |  |  |  |  |  |  |  |  |
| 1st quartile | 19,892 (22.1) | 20,018 (21.6) | 0.010 | -0.004 | 20,936 (22.1) | 25,417 (21.1) | 0.026 | -0.002 |
| 2nd quartile | 22,066 (24.5) | 23,183 (25.1) | -0.013 | 0.001 | 23,179 (24.5) | 30,379 (25.2) | -0.016 | 0.000 |
| 3rd quartile | 29,997 (33.3) | 31,362 (33.9) | -0.013 | 0.000 | 31,411 (33.2) | 41,362 (34.3) | -0.023 | 0.000 |
| 4th quartile | 18,200 (20.2) | 17,962 (19.4) | 0.019 | 0.003 | 19,192 (20.3) | 23,575 (19.5) | 0.018 | 0.001 |
| **Region, n (%)** |  |  |  |  |  |  |  |  |
| Metropolitan | 60,753 (67.4) | 63,249 (68.4) | -0.021 | -0.002 | 64,330 (67.9) | 81,344 (67.4) | 0.012 | -0.004 |
| Urban | 29,400 (32.6) | 29,267 (31.6) | 0.021 | 0.002 | 30,385(32.1) | 39,370 (32.6) | -0.011 | 0.004 |
| Rural | ^‡^ | 9 (0) | -0.010 | 0.001 | ^‡^ | 19 (0) | -0.013 | -0.002 |
| **Obstetric characteristics, n (%)** | |  |  |  |  |  |  |  |
| Nulliparity | 32,639 (36.2) | 30,021 (32.4) | 0.079 | 0.000 | 35,030 (37) | 41,105 (34.9) | 0.044 | 0.003 |
| Multiple gestation | 1,825 (2) | 1,418 (1.5) | 0.037 | -0.003 | 1,932 (2) | 1,760 (1.5) | 0.044 | -0.003 |
| **Comorbid conditions, n (%)** |  |  |  |  |  |  |  |  |
| Anxiety | 1,663 (1.8) | 1,851 (2) | -0.011 | -0.005 | 1,765 (1.9) | 2,100 (1.7) | 0.009 | -0.005 |
| Asthma | 5,904 (6.5) | 7,433 (8) | -0.057 | -0.003 | 6,240 (6.6) | 8,959 (7.4) | -0.033 | -0.004 |
| Depression | 1,409 (1.6) | 1,458 (1.6) | -0.001 | -0.003 | 1,501 (1.6) | 1,765 (1.5) | 0.010 | -0.005 |
| Type 1 and 2 diabetes | 1,115 (1.2) | 997 (1.1) | 0.015 | -0.003 | 1,228 (1.3) | 1,171 (1) | 0.031 | -0.002 |
| Epilepsy/seizures | 148 (0.2) | 139 (0.2) | 0.004 | -0.001 | 269 (0.3) | 273 (0.2) | 0.011 | -0.003 |
| Gastrointestinal diseases | 53,355 (59.2) | 54,269 (58.7) | 0.011 | -0.006 | 56,378 (59.5) | 66,658 (55.2) | 0.087 | -0.006 |
| Hypertension | 1,077 (1.2) | 1,081 (1.2) | 0.002 | -0.004 | 1,332 (1.4) | 1,430 (1.2) | 0.020 | -0.004 |
| Renal disease | 406 (0.5) | 434 (0.5) | -0.003 | -0.002 | 439 (0.5) | 559 (0.5) | 0.000 | -0.003 |
| Thyroid disorders | 8,288 (9.2) | 8,056 (8.7) | 0.017 | 0.001 | 10,566 (11.2) | 12,201 (10.1) | 0.034 | -0.001 |
| Alcohol or drug dependence | 120 (0.1) | 119 (0.1) | 0.001 | -0.003 | 145 (0.2) | 154 (0.1) | 0.007 | 0.001 |
| Tobacco dependence | ^‡^ | ^‡^ | 0.001 | -0.001 | ^‡^ | 5 (0) | -0.006 | -0.001 |
| Endometriosis | 673 (0.7) | 544 (0.6) | 0.019 | -0.001 | 743 (0.8) | 731 (0.6) | 0.022 | -0.001 |
| Polycystic ovarian syndrome | 826 (0.9) | 800 (0.9) | 0.005 | -0.002 | 895 (0.9) | 1,108 (0.9) | 0.003 | -0.003 |
| Respiratory infection | 79,264 (87.9) | 87,361 (94.4) | **-0.231** | 0.004 | 84,024 (88.7) | 113,782 (94.2) | **-0.199** | -0.007 |
| Inflammatory diseases | 960 (1.1) | 818 (0.9) | 0.018 | -0.001 | 1,072 (1.1) | 868 (0.7) | 0.043 | 0.000 |
| Pain | 1,672 (1.9) | 1,844 (2) | -0.010 | -0.005 | 1,814 (1.9) | 2,189 (1.8) | 0.008 | -0.004 |
| Fever | 3,840 (4.3) | 7,117 (7.7) | **-0.145** | -0.009 | 4,478 (4.7) | 9,898 (8.2) | **-0.142** | -0.005 |
| Migraine/headache | 8,781 (9.7) | 14,129 (15.3) | **-0.168** | -0.010 | 9,731 (10.3) | 19,429 (16.1) | **-0.173** | -0.004 |
| **Co-medication use, n (%)** |  |  |  |  |  |  |  |  |
| Antibiotics | 84,072 (93.3) | 86,344 (93.3) | -0.003 | -0.003 | 88,534 (93.5) | 112,269 (93) | 0.019 | -0.012 |
| Antiepileptics | 1,124 (1.2) | 1,026 (1.1) | 0.013 | -0.007 | 1,386 (1.5) | 1,291 (1.1) | 0.035 | -0.005 |
| Antidepressants | 3,066 (3.4) | 3,300 (3.6) | -0.009 | -0.008 | 3,236 (3.4) | 3,721 (3.1) | 0.019 | -0.009 |
| Antihypertensives | 2,944 (3.3) | 3,054 (3.3) | -0.002 | -0.007 | 3,184 (3.4) | 3,602 (3) | 0.022 | -0.006 |
| Antipsychotics | 450 (0.5) | 425 (0.5) | 0.006 | -0.003 | 509 (0.5) | 502 (0.4) | 0.018 | -0.003 |
| Anxiolytics | 20,397 (22.6) | 22,135 (23.9) | -0.031 | -0.010 | 21,248 (22.4) | 25,695 (21.3) | 0.028 | -0.011 |
| Azoles | 27,412 (30.4) | 26,951 (29.1) | 0.028 | -0.001 | 31,362 (33.1) | 38,096 (31.6) | 0.033 | -0.005 |
| Thyroid hormones | 2,919 (3.2) | 2,584 (2.8) | 0.026 | 0.002 | 3,443 (3.6) | 3,623 (3) | 0.035 | 0.000 |
| Fertility drugs | 4,586 (5.1) | 4,584 (5) | 0.006 | 0.003 | 5,017 (5.3) | 6,606 (5.5) | -0.008 | 0.001 |
| Hypnotics | 1,640 (1.8) | 1,800 (1.9) | -0.009 | -0.006 | 1,731 (1.8) | 1,826 (1.5) | 0.025 | -0.007 |
| Insulin | 584 (0.6) | 490 (0.5) | 0.015 | -0.003 | 711 (0.8) | 608 (0.5) | 0.031 | -0.002 |
| Non-insulin antidiabetic drugs | 561 (0.6) | 438 (0.5) | 0.020 | -0.004 | 585 (0.6) | 488 (0.4) | 0.030 | -0.003 |
| Lipid lowering drug | 526 (0.6) | 498 (0.5) | 0.006 | 0.000 | 555 (0.6) | 505 (0.4) | 0.024 | 0.002 |
| Opioid analgesics | 64,800 (71.9) | 70,804 (76.5) | **-0.106** | -0.009 | 68,712 (72.5) | 91,207 (75.5) | -0.068 | -0.009 |
| Antiacid | 69,293 (76.9) | 68,244 (73.8) | 0.072 | -0.007 | 72,662 (76.7) | 83,742 (69.4) | **0.166** | -0.004 |
| Corticosteroids | 50,506 (56) | 48,627 (52.6) | 0.070 | -0.006 | 53,355 (56.3) | 59,004 (48.9) | **0.150** | -0.005 |
| Triptans | 511 (0.6) | 643 (0.7) | -0.016 | -0.003 | 550 (0.6) | 687 (0.6) | 0.002 | -0.002 |
| Antiemetics | 18,118 (20.1) | 25,110 (27.1) | **-0.166** | -0.009 | 20,527 (21.7) | 34,234 (28.4) | **-0.155** | -0.007 |
| Medication for asthma/COPD | 25,777 (28.6) | 29,034 (31.4) | -0.061 | -0.005 | 27,071 (28.6) | 35,265 (29.2) | -0.014 | -0.006 |
| DMARD | 1,105 (1.2) | 971 (1) | 0.017 | 0.002 | 1,211 (1.3) | 1,206 (1) | 0.026 | 0.002 |
| Immunosuppressants | 5 (0) | 5 (0) | 0.000 | 0.000 | 5 (0) | 7 (0) | -0.001 | -0.001 |
| **General markers of burden of illness** | |  |  |  |  |  |  |  |
| OCI, mean (SD) | 0.8 (1) | 0.7 (1) | 0.083 | -0.006 | 0.8 (1) | 0.7 (1) | 0.094 | -0.007 |
| No. of distinct diagnoses, mean (SD) | 7.1 (4.6) | 7.3 (4.7) | -0.029 | -0.012 | 7 (4.6) | 6.8 (45) | 0.050 | -0.016 |
| No. of distinct prescription drugs, excluding NSAIDs, mean (SD) | 6.4 (4.9) | 6.8 (5.1) | -0.075 | -0.014 | 6.3 (4.8) | 6.3 (4.8) | 0.009 | -0.018 |
| Emergency room visits, n (%) | 8,132 (9) | 8,798 (9.5) | -0.017 | -0.008 | 8,481 (9) | 11,271 (9.3) | -0.013 | -0.008 |
| Patients hospitalized, n (%) | 7,904 (8.8) | 7,744 (8.4) | 0.014 | -0.005 | 8,276 (8.7) | 9,852 (8.2) | 0.021 | -0.003 |
| No. of outpatient visits, mean (SD) | 8.4 (7.8) | 8.6 (8.3) | -0.023 | -0.011 | 8.3 (7.7) | 7.7 (6.9) | 0.075 | -0.013 |
| No. of pregnancy-related hospital visits during first month, n (%) | 28,339 (31.4) | 32,001 (34.6) | -0.067 | 0.006 | 30,150 (31.8) | 45,898 (38) | **-0.130** | 0.004 |

**Abbreviation**: COPD=chronic obstructive pulmonary disease, DMARD=disease-modifying antirheumatic drugs, NSAID=non-steroidal anti-inflammatory drug, OCI=obstetric comorbidity index, PS=propensity score, SD=standard deviation, SMD=standardized mean difference

^*^To account for PS, the unexposed observations were weighted using the distribution of the exposed observations among 50 PS strata. Observations from the non-overlapping regions of the PS distributions were trimmed.

^†^Value >0.1 indicates significant imbalance between the exposed and unexposed groups.

^‡^Counts less than five are suppressed due to internal regulations of the National Health Insurance Service, South Korea
