# Supplementary material for: Neonatal and maternal adverse outcomes and exposure to nonsteroidal anti-inflammatory drugs during early pregnancy in South Korea: A nationwide cohort study
Source: PLoS Med. 2023 Feb 27;20(2):e1004183. doi: 10.1371/journal.pmed.1004183 (PMC9970080; doi:10.1371/journal.pmed.1004183)
Supplement: S4 Table — (DOCX) [file pmed.1004183.s005.docx]

**S4 Table.** Risk of neonatal and maternal adverse outcomes following early prenatal exposure to aspirin versus unexposed pregnancies

|  | **Aspirin** | | **Unexposed** | | **RR (95% CI)** | |
| --- | --- | --- | --- | --- | --- | --- |
|  | **Events/Total** | **Risk**  **/1,000 units^†^** | **Events/Total** | **Risk**  **/1,000 units^†^** | **Unadjusted** | **PS-adjusted** |
| Overall malformations | 549/11,820 | 46.45 | 55,406/1,757,268 | 31.53 | 1.47 (1.36-1.60) | 1.23 (1.13-1.34) |
| Low birth weight | 836/12,750 | 65.57 | 59,978/1,746,685 | 34.34 | 1.91 (1.79-2.04) | 1.22 (1.14-1.30) |
| Antepartum hemorrhage | 219/12,750 | 17.18 | 16,249/1,746,685 | 9.30 | 1.85 (1.62-2.11) | 1.04 (0.91-1.19) |
| Oligohydramnios | 121/12,750 | 9.49 | 10,760/1,746,685 | 6.16 | 1.54 (1.29-1.84) | 1.34 (1.12-1.51) |

**Abbreviation:** PS=propensity score, RR=relative risk.

^†^Units: births for outcomes of overall congenital malformations and low birth weights; pregnancies for outcomes of antepartum hemorrhage and oligohydramnios.
