# Supplementary material for: Neonatal and maternal adverse outcomes and exposure to nonsteroidal anti-inflammatory drugs during early pregnancy in South Korea: A nationwide cohort study
Source: PLoS Med. 2023 Feb 27;20(2):e1004183. doi: 10.1371/journal.pmed.1004183 (PMC9970080; doi:10.1371/journal.pmed.1004183)
Supplement: S5 Table — (DOCX) [file pmed.1004183.s006.docx]

**S5 Table.** Risk of congenital malformations in infants following maternal exposure to NSAID during the first trimester compared with unexposed pregnancies

|  | **NSAIDs (n=112,119)** | | **Unexposed (1,786,278)** | | **RD_1,000_^*^** | **RR (95% CI)** | |
| --- | --- | --- | --- | --- | --- | --- | --- |
|  | **Events** | **Risk/1,000 units^†^** | **Events** | **Risk/1,000 units^†^** |  | **Unadjusted** | **PS-adjusted** |
| Overall malformations | 4,583 | 40.88 | 56,681 | 31.73 | 9.15 | 1.29 (1.25-1.33) | 1.14 (1.10-1.18) |
| Nervous system | 249 | 2.22 | 3,647 | 2.04 | 0.18 | 1.09 (0.96-1.24) | 1.04 (0.90-1.21) |
| Eye | 110 | 0.98 | 1,559 | 0.87 | 0.11 | 1.12 (0.93-1.36) | 1.10 (0.88-1.39) |
| Ear, face, and neck | 33 | 0.29 | 367 | 0.21 | 0.08 | 1.43 (1.00-2.05) | 1.54 (1.02-2.33) |
| Heart defects | 2,988 | 26.65 | 34,055 | 19.06 | 7.59 | 1.40 (1.35-1.45) | 1.19 (1.13-1.24) |
| Respiratory system | 53 | 0.47 | 682 | 0.38 | 0.09 | 1.24 (0.94-1.64) | 1.28 (0.91-1.78) |
| Oral clefts | 162 | 1.44 | 2,233 | 1.20 | 0.24 | 1.16 (0.99-1.36) | 1.12 (0.92-1.36) |
| Digestive system | 307 | 2.74 | 3,847 | 2.15 | 0.59 | 1.27 (1.13-1.43) | 1.17 (1.02-1.35) |
| Abdominal wall defects | 18 | 0.16 | 221 | 0.12 | 0.04 | 1.30 (0.80-2.10) | 1.00 (0.55-1.78) |
| Urinary system | 610 | 5.44 | 8,401 | 4.70 | 0.74 | 1.16 (1.07-1.26) | 1.04 (0.94-1.15) |
| Genital organs | 164 | 1.46 | 2,391 | 1.34 | 0.12 | 1.09 (0.93-1.28) | 1.02 (0.85-1.23) |
| Limb | 190 | 1.69 | 2,487 | 1.39 | 0.30 | 1.22 (1.05-1.41) | 1.11 (0.93-1.32) |
| Others | 203 | 1.81 | 2,695 | 1.51 | 0.30 | 1.20 (1.04-1.38) | 1.19 (1.00-1.40) |

**Abbreviation:** NSAID=non-steroidal anti-inflammatory drug, PS=propensity score, RD=risk difference, RR=relative risk

^†^Units: births for outcomes of overall congenital malformations and low birth weights; pregnancies for outcomes of antepartum hemorrhage and oligohydramnios.

*RD_1,000_=Risk difference per 1,000 births.
