# Supplementary material for: Neonatal and maternal adverse outcomes and exposure to nonsteroidal anti-inflammatory drugs during early pregnancy in South Korea: A nationwide cohort study
Source: PLoS Med. 2023 Feb 27;20(2):e1004183. doi: 10.1371/journal.pmed.1004183 (PMC9970080; doi:10.1371/journal.pmed.1004183)
Supplement: S6 Table — (DOCX) [file pmed.1004183.s007.docx]

**S6 Table.** Risk of congenital malformations in infants following maternal exposure to NSAID during the first trimester compared with acetaminophen-exposed pregnancies

|  | **NSAIDs (n=90,155)** | | **Acetaminophen (n=92,525)** | | **RD_1,000_^*^** | **RR (95% CI)** | |
| --- | --- | --- | --- | --- | --- | --- | --- |
|  | **Events** | **Risk/1,000 units^†^** | **Events** | **Risk/1,000 units^†^** |  | **Unadjusted** | **PS-adjusted** |
| Overall malformations | 3,735 | 41.43 | 3,180 | 34.37 | 7.06 | 1.21 (1.15-1.26) | 1.15 (1.10-1.21) |
| Nervous system | 204 | 2.26 | 174 | 1.88 | 0.38 | 1.20 (0.98-1.47) | 1.16 (0.94-1.44) |
| Eye | 89 | 0.99 | 67 | 0.72 | 0.27 | 1.36 (0.99-1.87) | 1.28 (0.92-1.79) |
| Ear, face, and neck | 28 | 0.31 | 23 | 0.25 | 0.06 | 1.25 (0.72-2.17) | 1.42 (0.81-2.49) |
| Heart defects | 2,445 | 27.12 | 1,988 | 21.49 | 5.63 | 1.26 (1.19-1.34) | 1.17 (1.10-1.25) |
| Respiratory system | 43 | 0.48 | 44 | 0.48 | 0.001 | 1.00 (0.66-1.53) | 1.01 (0.65-1.58) |
| Oral clefts | 125 | 1.39 | 133 | 1.44 | -0.05 | 0.96 (0.76-1.23) | 0.96 (0.74-1.24) |
| Digestive system | 243 | 2.70 | 227 | 2.45 | 0.25 | 1.10 (0.92-1.32) | 1.08 (0.89-1.30) |
| Abdominal wall defects | 16 | 0.18 | 9 | 0.04 | 0.14 | 1.82 (0.81-4.13) | 1.73 (0.75-3.99) |
| Urinary system | 500 | 5.55 | 438 | 4.73 | 0.82 | 1.17 (1.03-1.33) | 1.15 (1.00-1.31) |
| Genital organs | 146 | 1.62 | 115 | 1.24 | 0.38 | 1.30 (1.02-1.66) | 1.20 (0.93-1.56) |
| Limb | 147 | 1.63 | 129 | 1.39 | 0.24 | 1.17 (0.92-1.48) | 1.12 (0.87-1.45) |
| Others | 168 | 1.86 | 158 | 1.71 | 0.15 | 1.09 (0.88-1.36) | 1.04 (0.83-1.31) |

**Abbreviation:** NSAID=non-steroidal anti-inflammatory drug, PS=propensity score, RD=risk difference, RR=relative risk

^†^Units: births for outcomes of overall congenital malformations and low birth weights; pregnancies for outcomes of antepartum hemorrhage and oligohydramnios.

*RD_1,000_=Risk difference per 1,000 births.
