# Supplementary material for: Neonatal and maternal adverse outcomes and exposure to nonsteroidal anti-inflammatory drugs during early pregnancy in South Korea: A nationwide cohort study
Source: PLoS Med. 2023 Feb 27;20(2):e1004183. doi: 10.1371/journal.pmed.1004183 (PMC9970080; doi:10.1371/journal.pmed.1004183)
Supplement: S7 Table — (DOCX) [file pmed.1004183.s008.docx]

**S7 Table.** Risk of congenital malformations in infants following maternal exposure to NSAID during the first trimester compared with NSAID past users

|  | **NSAIDs (n=112,119)** | | **Past users (n=353,724)** | | **RD_1,000_^*^** | **RR (95% CI)** | |
| --- | --- | --- | --- | --- | --- | --- | --- |
|  | **Events** | **Risk/1,000 units^†^** | **Events** | **Risk/1,000 units^†^** |  | **Unadjusted** | **PS-adjusted** |
| **Overall malformations** | 4,583 | 40.88 | 11,926 | 33.72 | 7.16 | 1.21 (1.17-1.25) | 1.16 (1.11-1.20) |
| Nervous system | 249 | 2.22 | 700 | 1.98 | 0.24 | 1.12 (0.97-1.30) | 1.09 (0.91-1.30) |
| Eye | 110 | 0.98 | 321 | 0.91 | 0.07 | 1.08 (0.87-1.34) | 1.17 (0.92-1.49) |
| Ear, face, and neck | 33 | 0.29 | 88 | 0.20 | 0.09 | 1.18 (0.79-1.77) | 1.00 (0.58-1.72) |
| Heart defects | 2,988 | 26.65 | 7,255 | 20.51 | 6.14 | 1.30 (1.25-1.36) | 1.19 (1.13-1.25) |
| Respiratory system | 53 | 0.47 | 157 | 0.44 | 0.03 | 1.07 (0.78-1.45) | 1.03 (0.73-1.47) |
| Oral clefts | 162 | 1.44 | 463 | 1.31 | 0.13 | 1.10 (0.92-1.32) | 1.13 (0.92-1.40) |
| Digestive system | 307 | 2.74 | 737 | 2.08 | 0.66 | 1.31 (1.15-1.50) | 1.22 (1.04-1.44) |
| Abdominal wall defects | 18 | 0.16 | 56 | 0.16 | 0.002 | 1.01 (0.60-1.72) | 1.34 (0.78-2.30) |
| Urinary system | 610 | 5.44 | 1,698 | 4.80 | 0.64 | 1.13 (1.03-1.24) | 1.10 (0.98-1.23) |
| Genital organs | 164 | 1.46 | 494 | 1.40 | 0.06 | 1.05 (0.88-1.25) | 1.06 (0.86-1.31) |
| Limb | 190 | 1.69 | 541 | 1.53 | 0.16 | 1.11 (0.94-1.31) | 1.10 (0.90-1.35) |
| Others | 203 | 1.81 | 611 | 1.73 | 0.08 | 1.05 (0.89-1.23) | 1.07 (0.89-1.28) |

**Abbreviation:** NSAID=non-steroidal anti-inflammatory drug, PS=propensity score, RD=risk difference, RR=relative risk

^†^Units: births for outcomes of overall congenital malformations and low birth weights; pregnancies for outcomes of antepartum hemorrhage and oligohydramnios.

*RD_1,000_=Risk difference per 1,000 births.
