# Supplementary material for: Neonatal and maternal adverse outcomes and exposure to nonsteroidal anti-inflammatory drugs during early pregnancy in South Korea: A nationwide cohort study
Source: PLoS Med. 2023 Feb 27;20(2):e1004183. doi: 10.1371/journal.pmed.1004183 (PMC9970080; doi:10.1371/journal.pmed.1004183)
Supplement: S8 Table — (DOCX) [file pmed.1004183.s009.docx]

**S8 Table.** Risk of neonatal and maternal adverse outcomes following exposure to NSAIDs during early pregnancy that restricted to pregnancies with severe respiratory infections

|  | **NSAIDs** | | **Unexposed** | | **RR (95% CI)** | |
| --- | --- | --- | --- | --- | --- | --- |
|  | **Events**  **/Total** | **Risk**  **/1,000 units^†^** | **Events**  **/Total** | **Risk**  **/1,000 units^†^** | **Unadjusted** | **PS-adjusted** |
| Overall malformations | 284/7,284 | 38.99 | 284/8,933 | 31.79 | 1.23 (1.04-1.44) | 1.08 (0.86-1.35) |
| Low birth weight | 428/8,474 | 50.51 | 427/13,107 | 32.58 | 1.55 (1.36-1.77) | 1.32 (1.09-1.60) |
| Antepartum hemorrhage | 92/8,474 | 10.86 | 89/13,107 | 6.79 | 1.60 (1.20-2.14) | 1.19 (0.83-1.71) |
| Oligohydramnios | 35/8,474 | 4.13 | 51/13,107 | 3.89 | 1.06 (0.69-1.63) | 1.30 (0.79-2.14) |

**Abbreviation:** CI=confidence interval, NSAID=non-steroidal anti-inflammatory drug, PS=propensity score, RR=relative risk

^†^Units: births for outcomes of overall congenital malformations and low birth weights; pregnancies for outcomes of antepartum hemorrhage and oligohydramnios.
