# Supplementary material for: Neonatal and maternal adverse outcomes and exposure to nonsteroidal anti-inflammatory drugs during early pregnancy in South Korea: A nationwide cohort study
Source: PLoS Med. 2023 Feb 27;20(2):e1004183. doi: 10.1371/journal.pmed.1004183 (PMC9970080; doi:10.1371/journal.pmed.1004183)
Supplement: S9 Table — (DOCX) [file pmed.1004183.s010.docx]

**S9 Table.** Risk of neonatal and maternal adverse outcomes following exposure to NSAIDs during early pregnancy that adjusted for severe respiratory infections

|  | **NSAIDs** | | **Unexposed** | | **RR (95% CI)** | |
| --- | --- | --- | --- | --- | --- | --- |
|  | **Events/Total** | **Risk**  **/1,000 units^†^** | **Events/Total** | **Risk**  **/1,000 units^†^** | **Unadjusted** | **PS-adjusted** |
| Overall malformations | 4,583/112,119 | 40.88 | 56,681/1,786,278 | 31.73 | 1.29 (1.25-1.33) | 1.14 (1.09-1.18) |
| Low birth weight | 6,798/124,957 | 54.40 | 61,574/1,770,891 | 34.77 | 1.56 (1.53-1.60) | 1.30 (1.26-1.34) |
| Antepartum hemorrhage | 1,699/124,957 | 13.60 | 16,645/1,770,891 | 9.40 | 1.45 (1.38-1.52) | 1.07 (1.01-1.14) |
| Oligohydramnios | 884/124,957 | 7.07 | 10,985/1,770,891 | 6.20 | 1.14 (1.07-1.22) | 1.12 (1.03-1.21) |

**Abbreviation:** CI=confidence interval, NSAID=non-steroidal anti-inflammatory drug, PS=propensity score, RR=relative risk

^†^Units: births for outcomes of overall congenital malformations and low birth weights; pregnancies for outcomes of antepartum hemorrhage and oligohydramnios.
