# Supplementary material for: Neonatal and maternal adverse outcomes and exposure to nonsteroidal anti-inflammatory drugs during early pregnancy in South Korea: A nationwide cohort study
Source: PLoS Med. 2023 Feb 27;20(2):e1004183. doi: 10.1371/journal.pmed.1004183 (PMC9970080; doi:10.1371/journal.pmed.1004183)
Supplement: S10 Table — (DOCX) [file pmed.1004183.s011.docx]

**S10 Table.** Risk of neonatal and maternal adverse outcomes following exposure to NSAIDs only in first trimester or early pregnancy

|  | **NSAIDs** | | **Unexposed** | | **RR (95% CI)** | |
| --- | --- | --- | --- | --- | --- | --- |
|  | **Events/Total** | **Risk**  **/1,000 units^†^** | **Events/Total** | **Risk**  **/1,000 units^†^** | **Unadjusted** | **PS-adjusted** |
| Overall malformations | 2,262/50,067 | 45.18 | 28,698/796,182 | 36.04 | 1.25 (1.20-1.31) | 1.14 (1.08-1.20) |
| Low birth weight | 3,437/57,844 | 59.42 | 33,960/802,461 | 42.32 | 1.40 (1.36-1.45) | 1.21 (1.16-1.27) |
| Antepartum hemorrhage | 866/57,844 | 14.97 | 9,442/802,461 | 11.77 | 1.27 (1.19-1.36) | 1.00 (0.92-1.09) |
| Oligohydramnios | 473/57,844 | 8.18 | 5,824/802,461 | 7.26 | 1.13 (1.03-1.24) | 1.13 (1.01-1.27) |

**Abbreviation:** CI=confidence interval, NSAID=non-steroidal anti-inflammatory drug, PS=propensity score, RR=relative risk

^†^Units: births for outcomes of overall congenital malformations and low birth weights; pregnancies for outcomes of antepartum hemorrhage and oligohydramnios.
