# Supplementary material for: Neonatal and maternal adverse outcomes and exposure to nonsteroidal anti-inflammatory drugs during early pregnancy in South Korea: A nationwide cohort study
Source: PLoS Med. 2023 Feb 27;20(2):e1004183. doi: 10.1371/journal.pmed.1004183 (PMC9970080; doi:10.1371/journal.pmed.1004183)
Supplement: S11 Table — (DOCX) [file pmed.1004183.s012.docx]

**S11 Table.** Risks of neonatal and maternal adverse outcomes following exposure to NSAIDs only in first trimester or early pregnancy: subgroup analysis according to cumulative duration of NSAIDs

|  | **NSAIDs** | **Unexposed** | **RR (95% CI)** | |
| --- | --- | --- | --- | --- |
|  | **Events**  **/Total** | **Events**  **/Total** | **Unadjusted** | **PS-adjusted** |
| **Overall malformations** |  |  |  |  |
| Cumulative duration (days) |  |  |  |  |
| <5 | 705/16,413 | 28,852/799,302 | 1.19 (1.11-1.28) | 1.10 (1.02-1.19) |
| 5-10 | 1,176/26,568 | 28,852/799,302 | 1.23 (1.16-1.30) | 1.12 (1.05-1.20) |
| >10 | 430/7,909 | 28,852/799,302 | 1.51 (1.37-1.65) | 1.30 (1.18-1.44) |
| **Low birth weight** |  |  |  |  |
| Cumulative duration (days) |  |  |  |  |
| <5 | 1,077/19,686 | 33,960/802,461 | 1.29 (1.22-1.37) | 1.14 (1.08-1.22) |
| 5-10 | 1,671/29,062 | 33,960/802,461 | 1.36 (1.30-1.43) | 1.20 (1.14-1.27) |
| >10 | 689/9,096 | 33,960/802,461 | 1.79 (1.66-1.92) | 1.42 (1.30-1.55) |
| **Antepartum hemorrhage** |  |  |  |  |
| Cumulative duration (days) |  |  |  |  |
| <5 | 270/19,686 | 9,442/802,461 | 1.17 (1.03-1.31) | 0.96 (0.84-1.09) |
| 5-10 | 456/29,062 | 9,442/802,461 | 1.33 (1.21-1.46) | 1.04 (0.94-1.16) |
| >10 | 140/9,096 | 9,442/802,461 | 1.31 (1.11-1.54) | 0.97 (0.81-1.16) |
| **Oligohydramnios** |  |  |  |  |
| Cumulative duration (days) |  |  |  |  |
| <5 | 160/19,686 | 5,824/802,461 | 1.12 (0.96-1.31) | 1.15 (0.97-1.35) |
| 5-10 | 235/29,062 | 5,824/802,461 | 1.11 (0.98-1.27) | 1.13 (0.98-1.31) |
| >10 | 78/9,096 | 5,824/802,461 | 1.18 (0.95-1.48) | 1.11 (0.87-1.42) |

**Abbreviation:** CI=confidence interval, NSAID=non-steroidal anti-inflammatory drug, PS=propensity score, RR=relative risk
