# Supplementary material for: Neonatal and maternal adverse outcomes and exposure to nonsteroidal anti-inflammatory drugs during early pregnancy in South Korea: A nationwide cohort study
Source: PLoS Med. 2023 Feb 27;20(2):e1004183. doi: 10.1371/journal.pmed.1004183 (PMC9970080; doi:10.1371/journal.pmed.1004183)
Supplement: S12 Table — (DOCX) [file pmed.1004183.s013.docx]

**S12 Table.** Risk of congenital malformations and low birth weight in infants following maternal exposure to NSAID during the first trimester according to three distinct referent group: sibling analyses^‡^

|  | **NSAIDs** | | **Reference** | | **RD_1,000_^*^** | **Odds Ratio**  **(95% CI)** |
| --- | --- | --- | --- | --- | --- | --- |
|  | **Events/Total** | **Risk/1,000 units^†^** | **Events/Total** | **Risk/1,000 units^†^** |  |  |
| **NSAIDs vs Unexposed** |  |  |  |  |  |  |
| Overall malformations | 1,016/27,224 | 37.32 | 877/28,709 | 30.55 | 6.77 | 1.13 (1.00-1.29) |
| Heart defects | 650/27,224 | 23.88 | 547/28,709 | 19.05 | 4.83 | 1.12 (0.94-1.32) |
| Digestive system | 68/27,224 | 2.50 | 50/28,709 | 1.74 | 0.76 | 1.29 (0.52-3.19) |
| Low birth weight | 1,330/29,855 | 44.55 | 1,039/31,437 | 33.05 | 11.50 | 1.30 (1.14-1.49) |
| **NSAIDs vs Acetaminophen** |  |  |  |  |  |  |
| Overall malformations | 107/2,878 | 37.18 | 104/2,896 | 35.91 | 1.27 | 1.18 (0.80-1.75) |
| Heart defects | 71/2,878 | 24.67 | 73/2,896 | 25.21 | -0.54 | 1.07 (0.64-1.80) |
| Urinary system | 16/2,878 | 5.56 | 11/2,896 | 3.8 | 1.76 | 1.32 (0.50-3.52) |
| Low birth weight | 146/3,457 | 42.23 | 142/3,482 | 40.78 | 1.45 | 0.84 (0.56-1.24) |
| **NSAIDs vs Past users** |  |  |  |  |  |  |
| Overall malformations | 387/10,447 | 37.04 | 343/10,575 | 32.43 | 4.61 | 1.03 (0.86-1.24) |
| Heart defects | 262/10,447 | 25.08 | 210/10,575 | 19.86 | 5.22 | 1.17 (0.93-1.48) |
| Digestive system | 29/10,447 | 2.78 | 20/10,575 | 1.89 | 0.89 | 1.42 (0.78-2.60) |
| Low birth weight | 384/8,469 | 45.34 | 294/8,520 | 34.51 | 10.83 | 1.35 (1.07-1.71) |

**Abbreviation:** NSAID=non-steroidal anti-inflammatory drug, PS=propensity score, RD=risk difference, RR=relative risk

^*^RD_1,000_=Risk difference per 1,000 births.

^†^Units: births for outcomes of overall congenital malformations and low birth weights; pregnancies for outcomes of antepartum hemorrhage and oligohydramnios.

^‡^Sibling analyses were conducted only for the neonatal outcomes that were significantly associated in the primary or secondary analyses (Figure 2, Supplementary Table 5-7).
