# Supplementary material for: Neonatal and maternal adverse outcomes and exposure to nonsteroidal anti-inflammatory drugs during early pregnancy in South Korea: A nationwide cohort study
Source: PLoS Med. 2023 Feb 27;20(2):e1004183. doi: 10.1371/journal.pmed.1004183 (PMC9970080; doi:10.1371/journal.pmed.1004183)
Supplement: S1 Fig — (DOCX) [file pmed.1004183.s014.docx]

**S1 Fig.** Propensity score distribution of NSAIDs-exposed and referent groups before and after propensity score based fine stratification.

|  |  | **PS distribution before PS fine stratification** | | **PS distribution after PS fine stratification** |
| --- | --- | --- | --- | --- |
| **NSAIDs vs.**  **Unexposed** | **Cohort 1** | 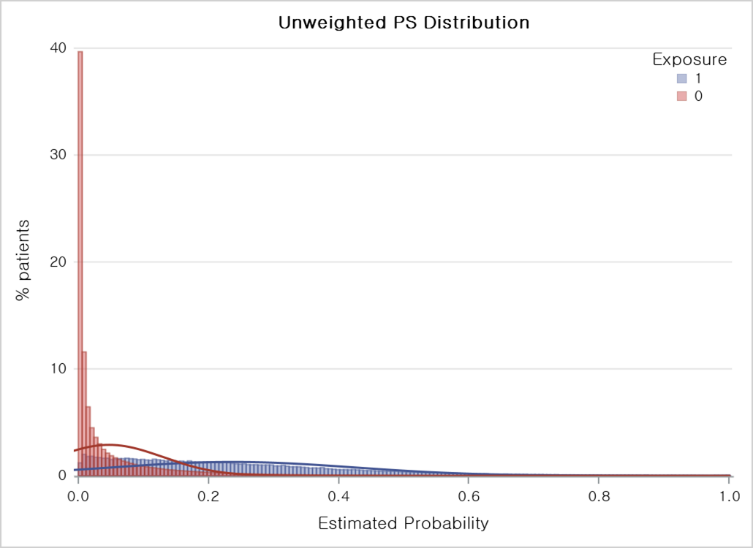 | | 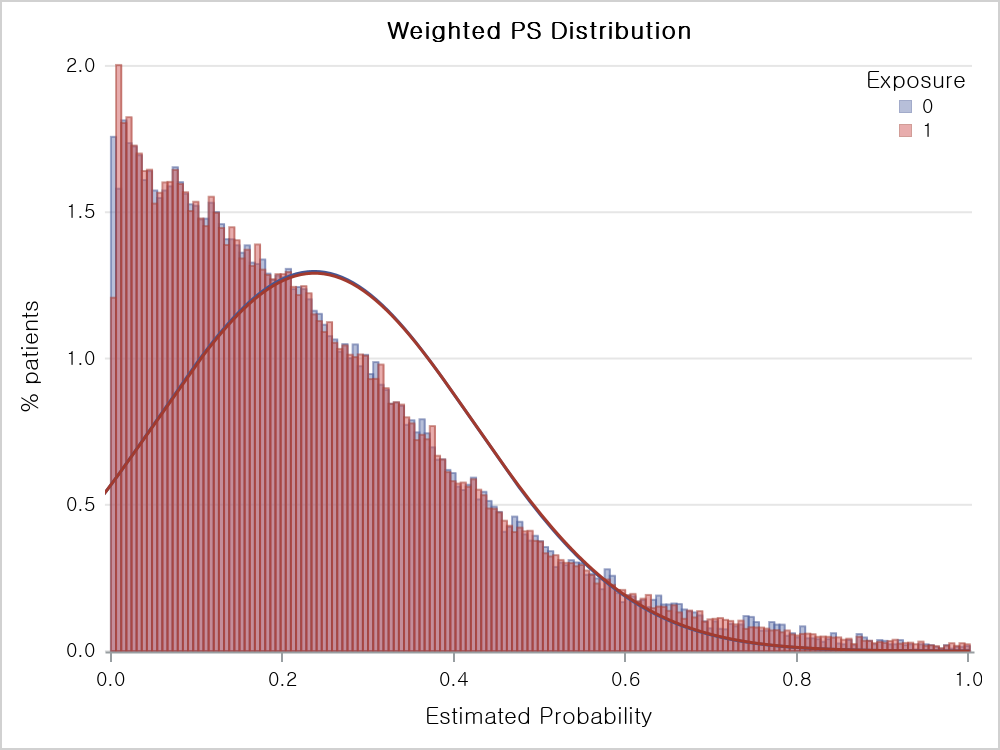 |
|  | **Cohort 2** | 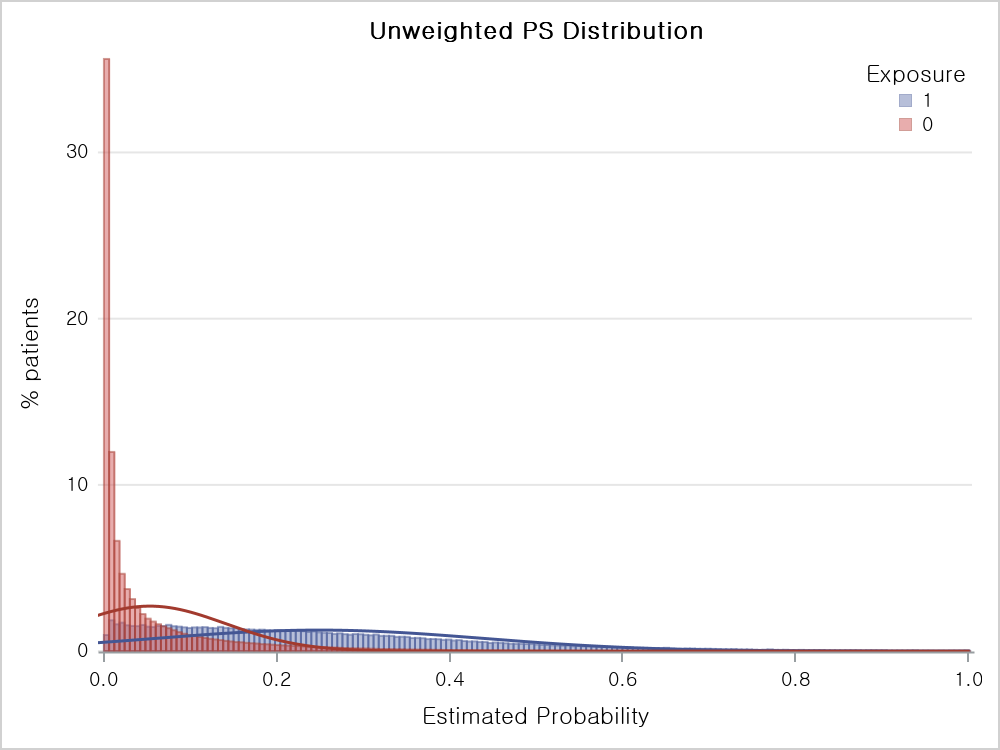 | | 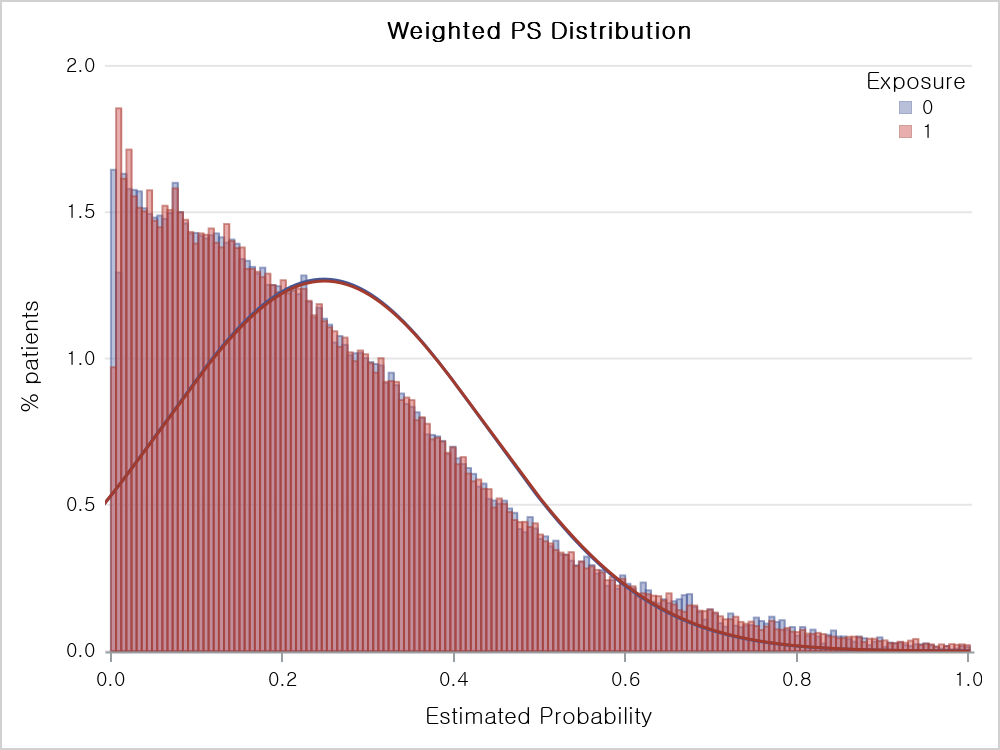 |
| **NSAIDs vs. Acetaminophen** | **Cohort 1** | 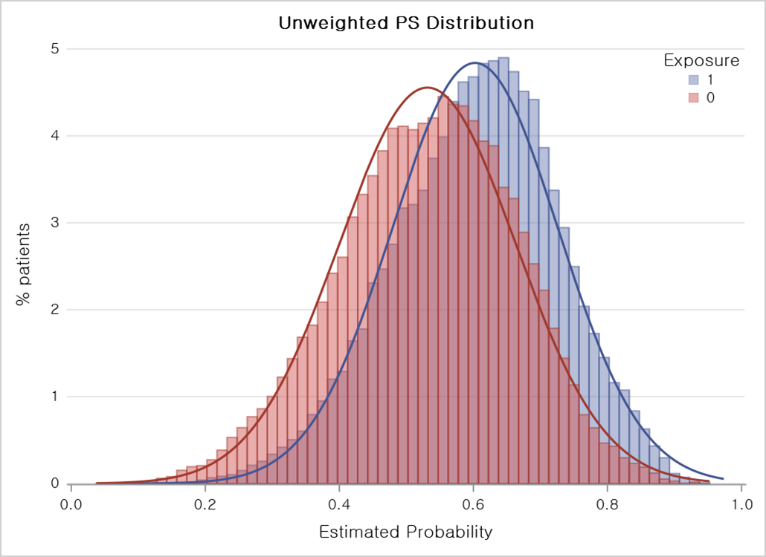 | 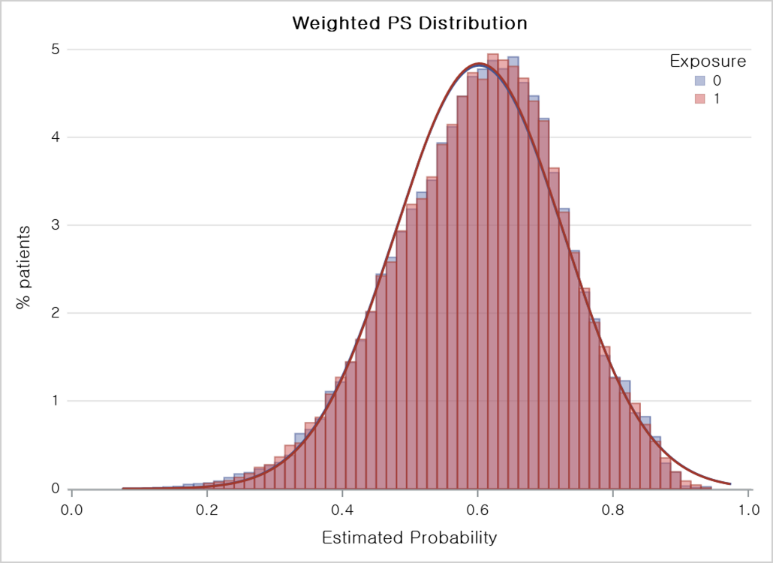 | |
|  | **Cohort 2** | 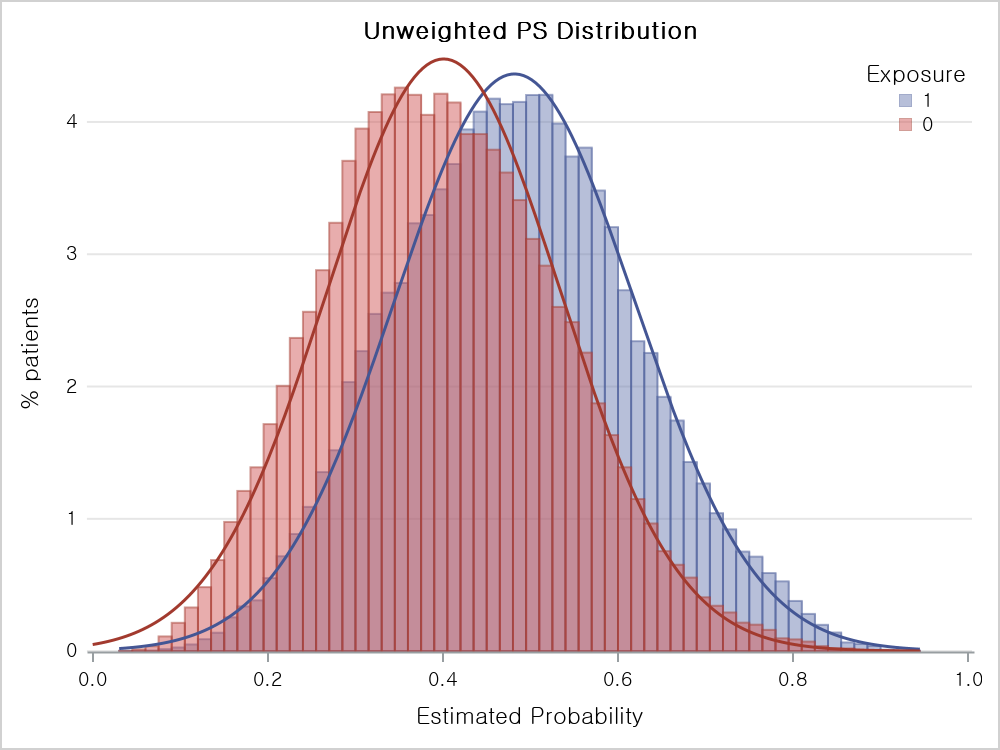 | 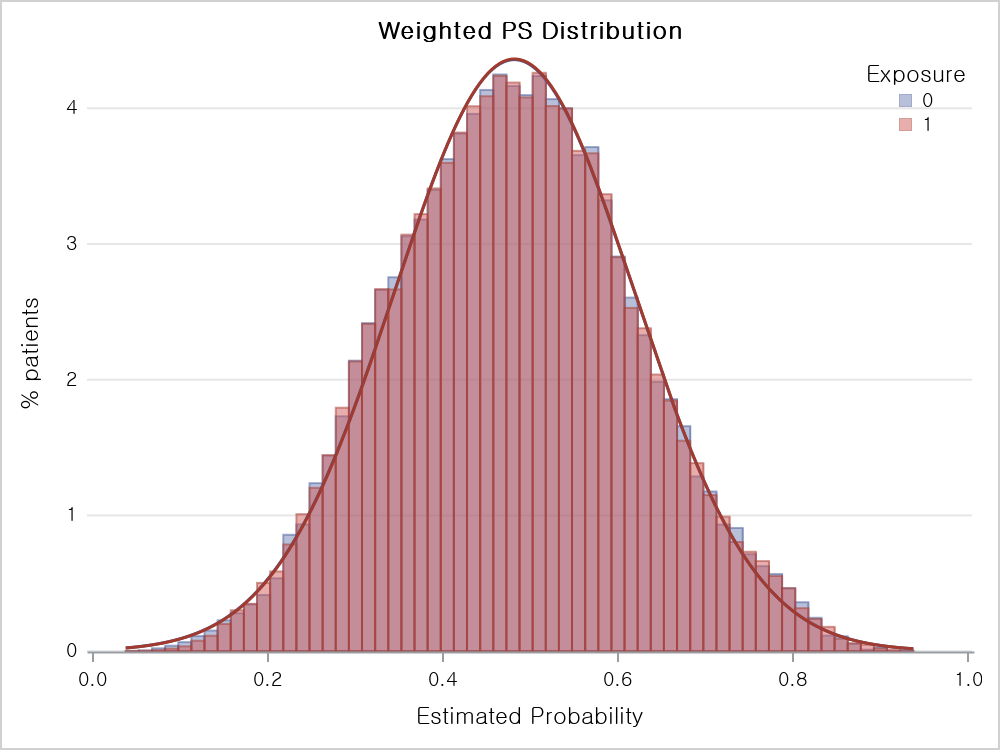 | |
| **NSAIDs vs.**  **Past users** | **Cohort 1** | 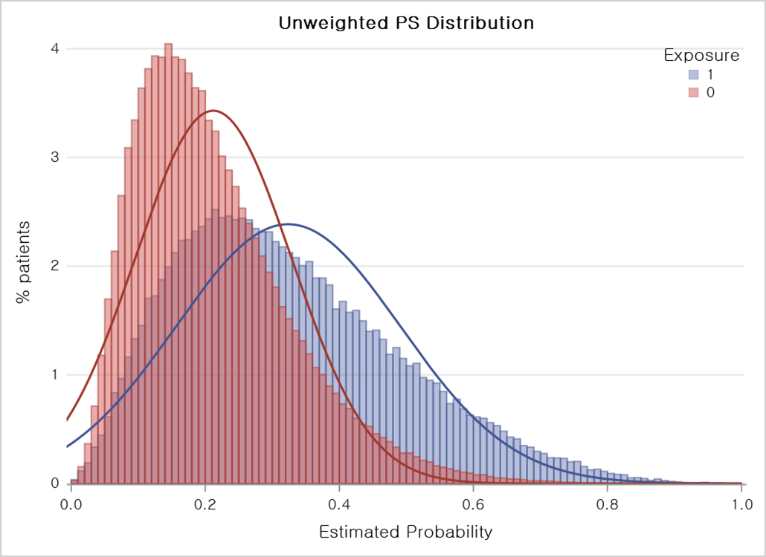 | 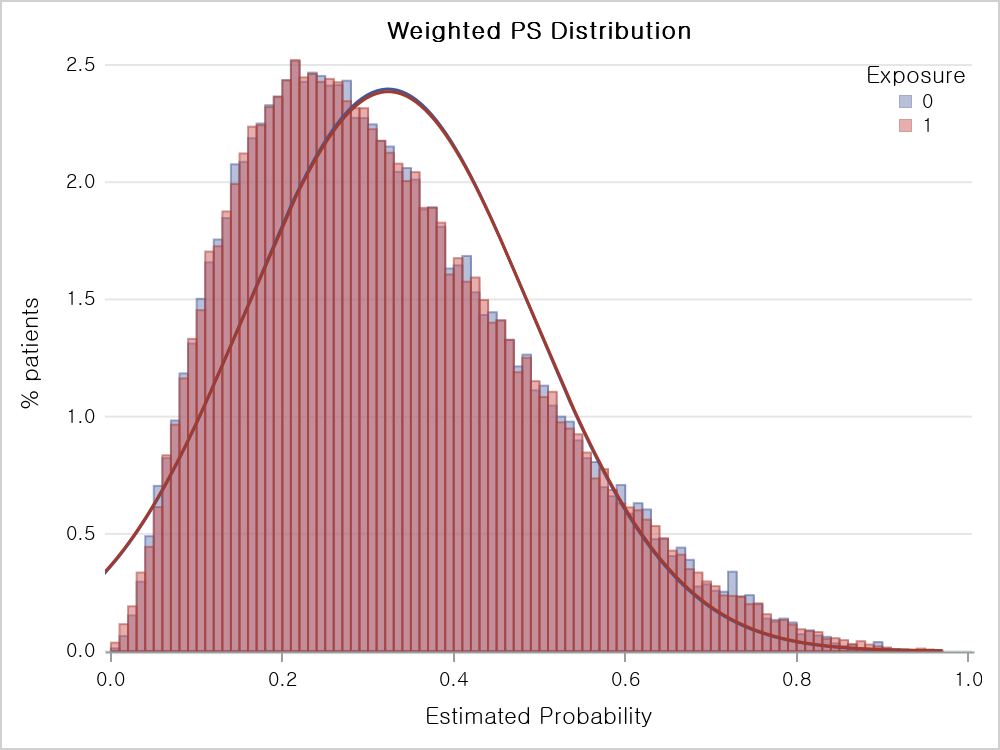 | |
|  | **Cohort 2** | 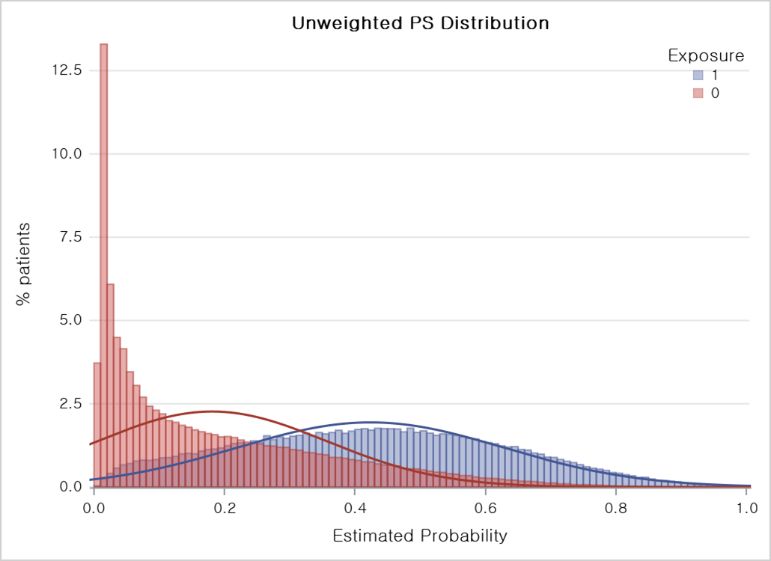 | 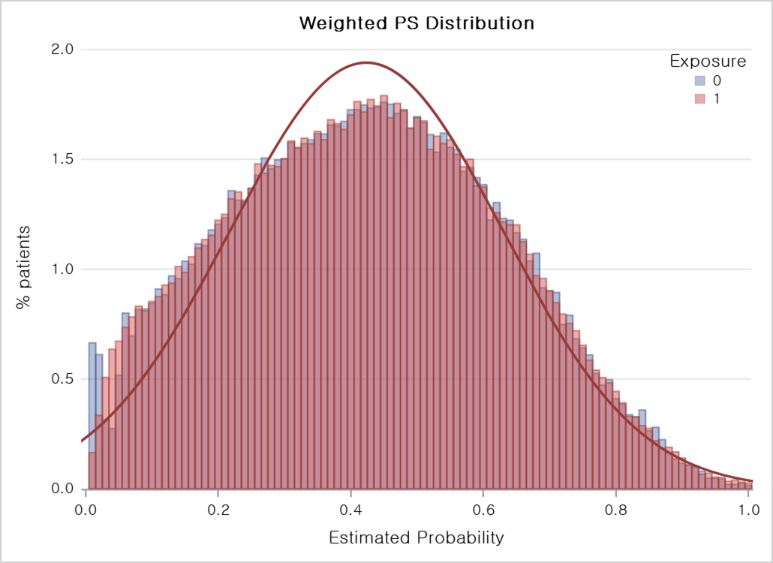 | |

**Abbreviation**: NSAID=non-steroidal anti-inflammatory drug, PS=propensity score.
