# Supplementary material for: Neonatal and maternal adverse outcomes and exposure to nonsteroidal anti-inflammatory drugs during early pregnancy in South Korea: A nationwide cohort study
Source: PLoS Med. 2023 Feb 27;20(2):e1004183. doi: 10.1371/journal.pmed.1004183 (PMC9970080; doi:10.1371/journal.pmed.1004183)

**S2 Fig.** Corrected relative risk for the association between NSAID exposure during the first trimester and overall congenital malformations starting from an observed RR of 1.14.


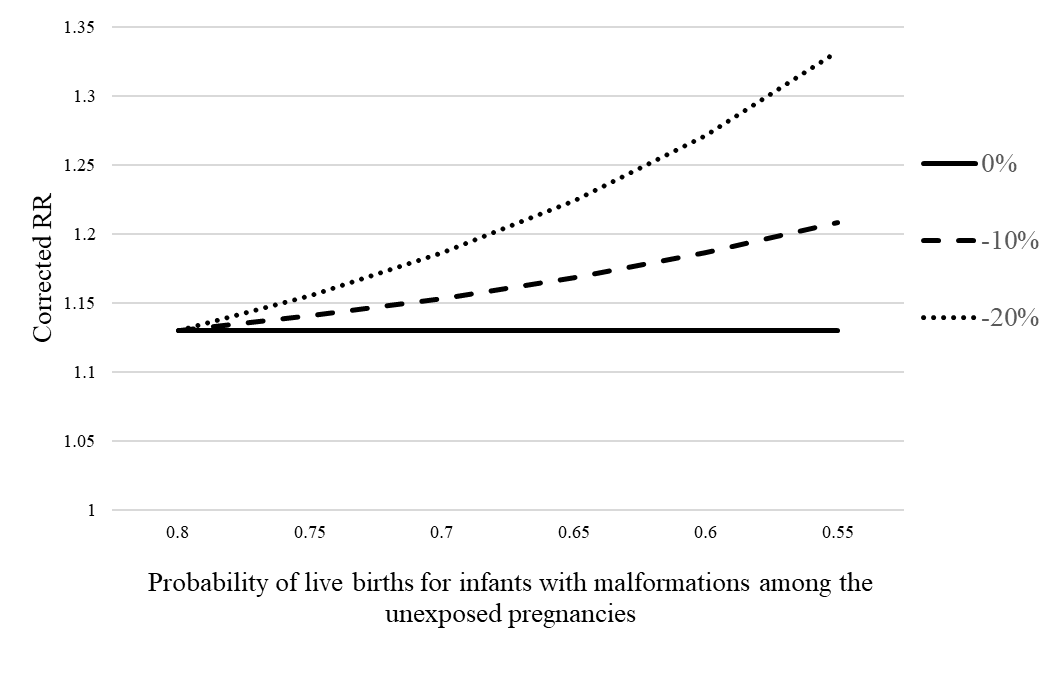

Supplement: S2 Fig — (DOCX) [file pmed.1004183.s015.docx]
