# Supplementary material for: Neonatal and maternal adverse outcomes and exposure to nonsteroidal anti-inflammatory drugs during early pregnancy in South Korea: A nationwide cohort study
Source: PLoS Med. 2023 Feb 27;20(2):e1004183. doi: 10.1371/journal.pmed.1004183 (PMC9970080; doi:10.1371/journal.pmed.1004183)
Supplement: S3 Fig — (DOCX) [file pmed.1004183.s016.docx]

**S3 Fig.** Corrected relative risk for the association between NSAID exposure during the first trimester and congenital heart defects starting from an observed RR of 1.19.


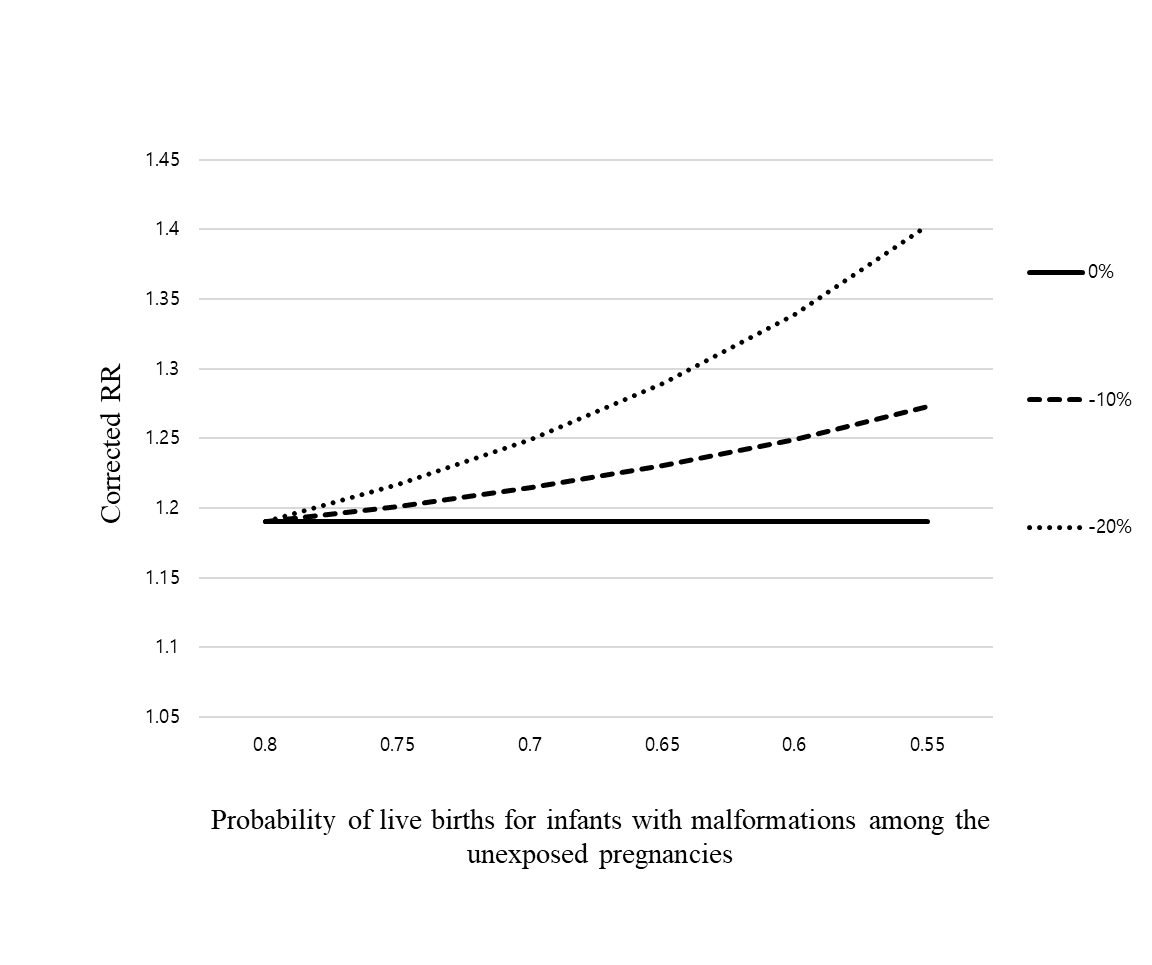


Supplementary Figures 3 and 4 show the corrected RR for overall congenital malformations and congenital heart defects, respectively. Based on the estimates in our main analysis (RR=1.14 and 1.19), the risk remained <1.34 for overall congenital malformations and <1.41 for congenital heart defects, respectively, under the most extreme scenario (assuming the selection probability of NSAIDs exposed pregnancies with malformation as 35%), which is highly unlikely.
