# Supplementary material for: Neonatal and maternal adverse outcomes and exposure to nonsteroidal anti-inflammatory drugs during early pregnancy in South Korea: A nationwide cohort study
Source: PLoS Med. 2023 Feb 27;20(2):e1004183. doi: 10.1371/journal.pmed.1004183 (PMC9970080; doi:10.1371/journal.pmed.1004183)
Supplement: S1 Appendix — (DOCX) [file pmed.1004183.s017.docx]

**SUPPLEMENTARY MATERIALS**

S1 Appendix. Potential effect of including live births only

S1 Appendix. Potential effect of including live births only

We included pregnancies that resulted in live births; did not include pregnancies ending in stillbirth or abortions. This may introduce selection bias when the probability of live birth differs between NSAID-exposed and unexposed pregnancies. For example, if the probability of live birth is lower in NSAID-exposed pregnancies than in unexposed pregnancies owing to a higher rate of pregnancy terminations due to severe malformations, then the estimates may be biased towards the null. Thus, we quantified the potential effects of missing non-live births. Considering the different probability of live births between NSAID-exposed and unexposed pregnancies, the corrected relative risks were estimated as below. This method has been widely used in previous studies to quantify the potential effect of restriction to live births ^1,2^:

Corrected RR=Observed RR*(S_10_*S_01_/S_11_*S_00_)

S_10_ refers to the probability of live births in NSAID-unexposed pregnancies with malformation.

S_01_ refers to the probability of live births in NSAID -exposed pregnancies without malformation.
S_11_ refers to the probability of live births in NSAID -exposed pregnancies with malformation.

S_00_ refers to the probability of live births in NSAID -unexposed pregnancies without malformation.

Based on estimates from the literature, the live birth probability among NSAID-unexposed pregnancies without malformation (S_00_) was defined as 80%.^3^ We then assumed the probability of live birth among unexposed pregnancies with malformations (S_10_) as a range of 55% to 80%, based on a previous study.^4^ Lastly, we evaluated the potential effect of lower frequency of live births, ranging from 10% to 20%, in NSAID exposed pregnancies.

eTable. Probability of live births

| **Pregnancies with malformations** | | **Pregnancies without malformations** | |
| --- | --- | --- | --- |
| **Unexposed (S**_10_**)** | **NSAID exposed (S**_11_**)** | **Unexposed (S**_00_**)** | **NSAID exposed (S**_01_**)** |
| 55–80% | S_10_-20% | 80% | S_00_-20% |
|  | S_10_-10% |  | S_00_-10% |
|  | S_10_ |  | S_00_ |

**References**

1. Huybrechts KF, Palmsten K, Avorn J, et al. Antidepressant use in pregnancy and the risk of cardiac defects. New England Journal of Medicine 2014;370(25):2397-407.

2. Patorno E, Huybrechts KF, Bateman BT, et al. Lithium use in pregnancy and the risk of cardiac malformations. New England Journal of Medicine 2017;376(23):2245-54.

3. Lee S-Y. 2,018 National Survey on Fertility, Family Health and Welfare: Korea Institute for Health and Social Affairs, 2019.

4. Svensson E, Ehrenstein V, Nørgaard M, et al. Brief Report: Estimating the Proportion of All Observed Birth Defects Occurring in Pregnancies Terminated by a Second-trimester Abortion. Epidemiology 2014:866-71.
