# Supplementary material for: Neonatal and maternal adverse outcomes and exposure to nonsteroidal anti-inflammatory drugs during early pregnancy in South Korea: A nationwide cohort study
Source: PLoS Med. 2023 Feb 27;20(2):e1004183. doi: 10.1371/journal.pmed.1004183 (PMC9970080; doi:10.1371/journal.pmed.1004183)
Supplement: S1 Protocol — (DOCX) [file pmed.1004183.s018.docx]

**SUPPLEMENTARY MATERIALS**

**S1 Protocol.** Summary protocol.

**S1 Protocol.** Summary Protocol

| **Protocol Title:** | Neonatal and Maternal Adverse Outcomes Following Exposure to Non-steroidal Anti-inflammatory Drugs During Early Pregnancy |
| --- | --- |
| **Study Objective:** | To investigate into the association of neonatal and maternal adverse outcomes with early prenatal exposure to non-steroidal anti-inflammatory drugs (NSAIDs). |
| **Study type:** | Observational |
| **Study Design:** | A retrospective nationwide cohort study using the National Health Insurance Service (NHIS) database of South Korea, which covers the entire South Korean population. |
| **Study Population:** | Pregnant women who gave birth in South Korea between April 2010 and December 2018  **Sex/Gender:** Female  **Ages:** 18 to 44 years (adult)  **Inclusion Criteria:**   - Pregnancies with live birth, 2010-2018 - Pregnancies linked to liveborn infants - Pregnancies aged 18-44 years at delivery   **Exclusion Criteria for Cohort 1:**   - Pregnancies with exposure to known teratogenic drugs (e.g. antineoplastic agent, warfarin, lithium, systemic retinoids, misoprostol, thalidomide, androgens, antiepileptic medications) during the first trimester - Infants with chromosomal abnormalities, genetic syndromes, and malformation syndromes with known causes - Pregnancies with no NSAID prescription during the first trimester, but with at least one NSAID prescription during the 3 months before the pregnancy onset - Pregnancies with only 1 NSAID prescription during the first trimester   **Exclusion Criteria for Cohort 2:**   - Pregnancies with no NSAID prescription during early pregnancy, but with at least one NSAID prescription within 3 months before the pregnancy onset - Pregnancies with only 1 NSAID prescription during early pregnancy |
| **Intervention:** | Exposure to NSAID during the first trimester or early pregnancy  **Groups:**  1) Pregnancies with exposure to NSAIDs:  Women receiving at least two NSAID prescription during the first trimester (first 90 days of pregnancy) or early pregnancy (first 19 weeks of pregnancy)  2) Pregnancies without exposure to NSAIDs:  Women who did not receive a NSAID prescription during the 3 months before the pregnancy onset through the end of the first trimester or early pregnancy |
| **Sample Size:** | Based on the estimated number of live births during 2010-2018 from the Korean Statistical Information Service (KOSIS), we anticipated that our study cohort would include approximately 3 million pregnancies in this study. |
| **Study Endpoints:** | Risk of neonatal outcomes of congenital malformations and low birth weight   - Overall and organ-specific malformations in infants confirmed by diagnostic records of infants in the first year of life in NHIS database - Low birth weight confirmed by diagnostic records in NHIS database   Risk of maternal outcomes of antepartum hemorrhage and oligohydramnios   - Antepartum hemorrhage and oligohydramnios confirmed by diagnostic records of mothers in NHIS database |
| **Statistical Methods:** | **Primary analysis:**   - Evaluate balance of characteristics between groups using an absolute standardized mean difference (aSMD); value >0.1 indicates a significant imbalance. - Estimate absolute risks (per 1,000 pregnancies), risk differences, and unadjusted relative risks (RR) with 95% confidence interval (CI) for risk of neonatal and maternal outcomes, stratified by exposure to NSAIDs. - Apply propensity score (PS) fine stratification weight method to control for potential confounders and the surrogates of potential confounders. - Estimate PS-adjusted RR with 95% CI using a weighted generalized linear model (log-binomial).   **Secondary analysis:**   - Three pre-specified secondary analyses: - Alternate referent groups (acetaminophen-exposed, discontinuers) - Type and individual ingredients of NSAIDs - Duration-response relationship   **Sensitivity analysis:**   1. Restrict cohort to nulliparous women to account for intra-individual correlations. 2. Restrict cohort to singleton pregnancies to eliminate the potential confounding effect of multiple gestations. 3. Restrict cohort to pregnant women with data on health examination records to assess residual confounding effects from body mass index and smoking status. 4. Restrict cohort to pregnant women who had underlying comorbidities related to indications for NSAIDs (e.g., inflammatory disease, respiratory infection, fever, pain, migraine/headache) to mitigate confounding by indication. 5. Redefined exposure assessment window as 4^th^ to 10^th^ week of gestational period for analysis of malformations, a previously reported duration of organogenesis (cohort 1 only). 6. Negative control analysis for all outcomes by ascertaining exposure between 5 and 8 months before the LMP; null finding indicates that the main findings were unaffected by residual confounding. 7. Sibling-matched analyses using conditional logistic regression models to estimate odds ratios for neonatal outcomes to address potential confounding from within-family shared factors. 8. Quantitative bias analysis based on the probabilistic method to address the impact of selection bias as the cohort included live births only. |
| **Contacts and Locations:** | **Principal Investigator:** Ju-Young Shin, PhD, Sungkyunkwan University  **Locations:** Sungkyunkwan University, Suwon, Gyeonggi-do 16419 Republic of Korea  **Sponsors and Collaborators:** National Research Foundation of Korea |
